# The wax gourd genomes offer insights into the genetic diversity and ancestral cucurbit karyotype

**DOI:** 10.1038/s41467-019-13185-3

**Published:** 2019-11-14

**Authors:** Dasen Xie, Yuanchao Xu, Jinpeng Wang, Wenrui Liu, Qian Zhou, Shaobo Luo, Wu Huang, Xiaoming He, Qing Li, Qingwu Peng, Xueyong Yang, Jiaqing Yuan, Jigao Yu, Xiyin Wang, William J. Lucas, Sanwen Huang, Biao Jiang, Zhonghua Zhang

**Affiliations:** 1 Guangdong Key Laboratory for New Technology Research of Vegetables, Vegetable Research Institute, Guangdong Academy of Agricultural Sciences, Guangzhou, Guangdong 510640 China; 2 Key Laboratory of Biology and Genetic Improvement of Horticultural Crops of the Ministry of Agriculture, Sino-Dutch Joint Laboratory of Horticultural Genomics, Institute of Vegetables and Flowers, Chinese Academy of Agricultural Sciences, Beijing, 100081 China; 3 Agricultural Genomic Institute at Shenzhen, Chinese Academy of Agricultural Sciences, Shenzhen, 518124 China; 4 School of Life Sciences, North China University of Science and Technology, Caofeidian Dist., Tangshan, Hebei 063200 China; 5 Department of Plant Biology, University of California, Davis, CA USA; 6 College of Horticulture, Qingdao Agricultural University, Qingdao, 266109 China

**Keywords:** Evolutionary genetics, Comparative genomics, Genetic variation, Natural variation in plants

## Abstract

The botanical family Cucurbitaceae includes a variety of fruit crops with global or local economic importance. How their genomes evolve and the genetic basis of diversity remain largely unexplored. In this study, we sequence the genome of the wax gourd (*Benincasa hispida*), which bears giant fruit up to 80 cm in length and weighing over 20 kg. Comparative analyses of six cucurbit genomes reveal that the wax gourd genome represents the most ancestral karyotype, with the predicted ancestral genome having 15 proto-chromosomes. We also resequence 146 lines of diverse germplasm and build a variation map consisting of 16 million variations. Combining population genetics and linkage mapping, we identify a number of regions/genes potentially selected during domestication and improvement, some of which likely contribute to the large fruit size in wax gourds. Our analyses of these data help to understand genome evolution and function in cucurbits.

## Abstract (web summary)

Cucurbits fruits have diverse shapes and sizes, but their genomes evolution and genetic basis of diversity are unclear. Here, the authors show that the wax gourd genome has the most ancestral karyotype among cucurbits and identify candidate genes which contribute to large fruit size by comparative and population genomics analyses.

## Introduction

The family Cucurbitaceae (cucurbits) includes numerous economically important species, including those bearing edible and medicinal fruits, such as cucumber (*Cucumis sativus*), melon (*Cucumis melo*), watermelon (*Citrullus lanatus*), bottle gourd (*Lagenaria siceraria*), wax gourd (*Benincasa hispida*), pumpkin, and squash (*Cucurbita* spp.). Although these species are monophyletic, they display fascinating phenotypic variation in fruit characteristics. Comparing a few available genomes at that time for cucumber^1^, melon^2^, watermelon^3^, and bottle gourd^4^ previously proposed that the 12 chromosomes of melon may represent the ancestral karyotype of the cucurbit species^4^. Some other species, such as wax gourd from the genus *Benincasa* and chayote (*Sechium edule*) from the tribe Sicyeae, also have 12 chromosomes. Whether these species have a similar karyotype, and whether they represent the ancestor of cucurbits remain to be determined. How these genomes evolve from their common ancestor remains to be resolved and requires analysis of the genomes of more cucurbit species.

Wax gourd, also known as ash gourd, white pumpkin, and white gourd, originated from the Indo-China region^5^ and is widely cultivated in India, Japan, China, and many other tropical areas, with increasing popularity in the Caribbean and the United States. Wild wax gourd has a small fruit (<10 cm in length), whereas most wax gourd cultivars bear a giant fruit (up to 80 cm in length and weight of over 20 kg). Its fruit contains important nutrients, such as vitamins and flavonoids^6^, and metabolites that can be used in treating various disorders^7,8^.

The wax gourd is the only member of the genus *Benincasa* in the tribe *Benincaseae*, which includes cucumber, melon, watermelon, and bottle gourd. This situation, along with its large genome size^9^ and 12 chromosomes, as in melon, makes it an excellent system for exploring the evolution of cucurbit genomes. Currently, only a high-density genetic map^10^, transcriptome sequences for several tissues^11^, and a small number of genomic fragments^12,13^ have been developed for wax gourd. Hence, an assembled wax gourd genome is needed to facilitate a further investigation of cucurbit genome evolution and to explore the genetic basis of its diversity.

Here, we report a high-quality draft genome assembly of wax gourd cultivar B227, which is used to explore the genome evolution of cucurbit species, revealing their ancestral genome. Furthermore, by combining population analyses and genetic dissection, we identify genomic regions and genes, which may be involved in the determination of wax gourd fruit size. This wax gourd genome sequence and the genomic variation map offer valuable resources not only for facilitating wax gourd genetic research and improvement but also for studying evolution and speciation in the cucurbits.

## Results

### Genome assembly and main features

An inbred wax gourd line, B227, bearing large mature fruit (up to 80 cm in length) with dark green skin, was selected for genome sequencing, using Illumina and single-molecule real-time (SMRT) sequencing technologies. A total of 55.4 Gb of high-quality, cleaned sequences were generated (Supplementary Table 1), representing ~50-fold coverage of the estimated 1.03 Gb genome, based on k-mer analysis of the Illumina sequences (Supplementary Fig. 1). Combing de novo assembly of Illumina and PacBio sequences yielded a draft genome of 913 Mb, with a scaffold N50 of 3.4 Mb length and longest scaffold of 14.5 Mb (Table 1, Supplementary Tables 2 and 3). Of the assembly, 859 Mb (94.1%), including 397 scaffolds, could be anchored to the 12 linkage groups, using the high-density genetic map^10^ (Supplementary Fig. 2a). Marker order on the genetic map was significantly consistent with that on the genome assembly (*p* = 0.995–0.999, Pearson’s correlation coefficient; Supplementary Fig. 3), indicating the high degree of accuracy of the assembly.

**Table 1:** Summary of wax gourd genome assembly and annotation

Estimated genome size (Gb)	1.03
Total length of scaffolds (Mb)	913
Number of scaffolds	2197
Longest scaffolds (Mb)	14.5
N50 of scaffolds (Mb)	3.4
N90 of scaffolds (Mb)	0.9
Anchored to chromosome (Mb)	859
No. of predicted protein-coding genes	27,467
Average gene length (bp)	3962
Masked repeat sequence length (Mb)	689.5
Percentage of repeat sequences (%)	75.5

The completeness of the wax gourd genome assembly was assessed using the BUSCO gene set^14^ and available RNA-sequencing (RNA-seq) data^11^. BUSCO analysis revealed that 93.2% of the core eukaryotic genes are present in the wax gourd genome, and 91.0% of them had complete coverage. In addition, 110 Gb of RNA-seq data obtained from five major tissue types (root, stem, young leaf, flower, and fruit) were mapped onto the wax gourd genome assembly. Overall, 94.6% of the RNA-seq reads could be mapped to the assembly. This extensive coverage of core eukaryotic genes, in conjunction with the high mapping rate of RNA-seq reads, indicated the high quality and overall completeness of the assembled genome.

A total of 689.5 Mb (75.5%) repetitive sequences and 27,467 protein-coding genes were predicted in the wax gourd genome (Table 1, Supplementary Table 4, Supplementary Figs. 2b and 4), Of these genes, 21,227 (77.28%) were annotated using known proteins (Supplementary Table 5), and 19,972 (72.71%) could be supported by RNA-seq data from sampled tissues^11^.

### Identification of the cucurbit ancestral genome

**Fig. 1:**
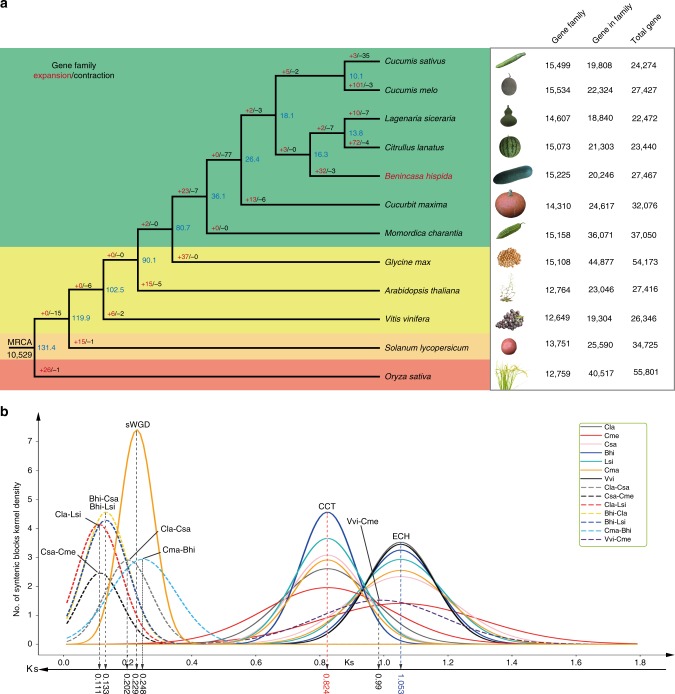
Phylogenetic relationship and comparative genomics analyses. **a** Phylogenetic tree of 12 plant species and evolution of gene families. Blue numerical value beside each node shows the estimated divergence time of each node (MYA, million years ago). Gene family: numbers of gene families in each species; gene in family: the numbers of genes that could be clustered into gene families; total gene: the total gene numbers for each species. **b** Distribution of synonymous substitution levels (Ks) of syntenic orthologous (solid curves) and paralogous genes (dashed curves) after evolutionary rate correction. Bhi: *Benincasa hispida*; Cma: *Cucurbit maxima*; Cme: *Cucumis melo*; Lsi: *Lagenaria siceraria*; Cla: *Citrullus lanatus*; Csa: *Cucumis sativus*; Vvi: *Vitis vinifera*. The source data underlying **a** is provided as a Source Data file

To explore the genome evolution of wax gourd, genes from the seven cucurbits (wax gourd, cucumber, melon, watermelon, bottle gourd, pumpkin, and bitter gourd), three rosid species (soybean, *Arabidopsis*, and grape), one asterid (tomato), and one monocot (rice) were clustered into 28,232 gene families. Of these, 463 single-copy gene families were determined and used to reconstruct a maximum-likelihood phylogenetic tree (Fig. 1a). This revealed that wax gourd and the ancestor of watermelon and bottle gourd, which diverged about 16.3 million years ago (MYA), form a sister clade to *Cucumis* species, and the two clades diverged about 18.1 MYA, consistent with an earlier report^15^. The tribe *Benincaseae* was estimated to have diverged from the tribe *Cucurbiteae*, containing squash, and the tribe *Momordiceae*, containing bitter gourd, 26.4 and 36.1 MYA, respectively. In wax gourd, 32 gene families comprising 324 genes exhibited significant expansions (*p* < 0.01) relative to their ancestor (Supplementary Data 1). Some of these families were annotated as cytochrome b–c1 complex subunit, zinc-finger protein, and NBS-LRR resistance genes (Supplementary Table 6, Supplementary Fig. 5). These genes might be a resource for investigating the specific features of wax gourd.

Whole-genome duplication (WGD) is thought to be a major driving force in evolution, as it provides additional genetic material that is then subject to divergence, sub-functionalization, and neofunctionalization^16–18^. To investigate WGD events in wax gourd, we identified syntenic blocks within its genome. The synonymous substitution rate (Ks) of collinear gene pairs indicated no recent WGD in wax gourd, and both the Eudicot-common hexaploidy (ECH)^19^ and the ancient cucurbit-common tetraploidization (CCT)^20^ events were observed (Fig. 1b, Supplementary Fig. 6a, b). This situation is similar to other species of the tribe *Benincaseae*, including cucumber^1^, melon^2^, watermelon^3^, and bottle gourd^4^, but distinct from pumpkin and squash genomes^21^ in the tribe *Cucurbiteae* with a recent WGD (Fig. 1b). In addition, the Ks distribution also suggests divergent evolutionary rates for these cucurbit species (Supplementary Fig. 6a). After correction using grape, as a reference to eliminate the influence of the ECH event, wax gourd and melon appear to have the slowest evolutionary rates (Supplementary Fig. 6b). Thus, the wax gourd genome has no recent WGD and has the slowest evolutionary rate among the cucurbits.

**Fig. 2:**
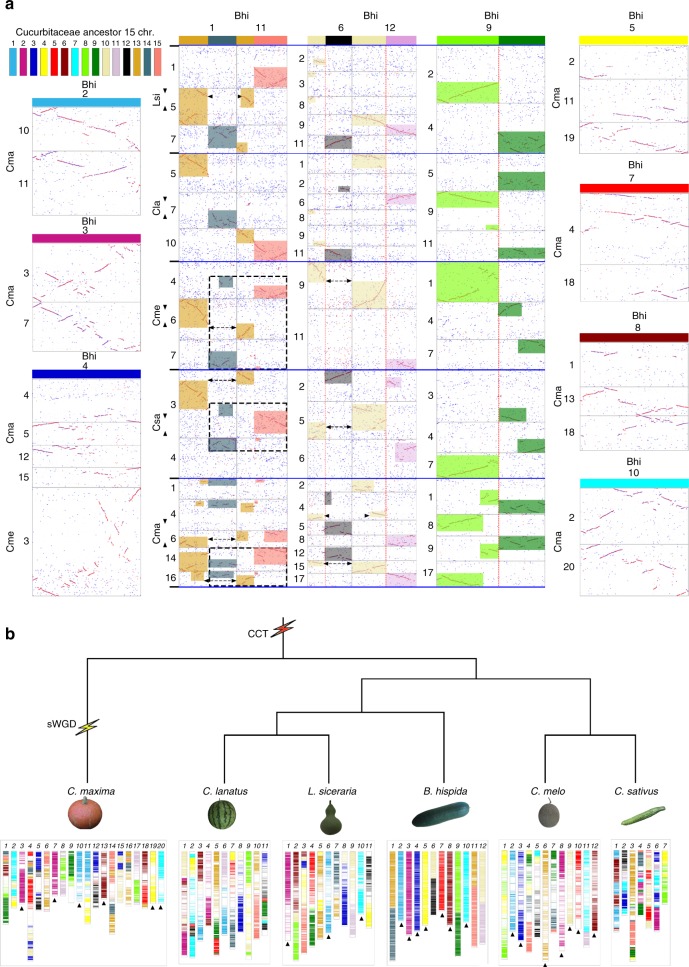
Genome evolution of the six sequenced cucurbit species. **a** Conserved genome karyotype of wax gourd and identification of ancestral chromosomes of cucurbit species. Different colors along the 12 wax gourd chromosomes indicate the origin of the 15 ancestral chromosomes. Bhi: *Benincasa hispida*; Cma: *Cucurbit maxima*; Cme: *Cucumis melo*; Lsi: *Lagenaria siceraria*; Cla: *Citrullus lanatus*; Csa: *Cucumis sativus*. **b** Evolutionary scenario of the Cucurbitaceae genomes from the ancestral Cucurbitaceae karyotype. sWGD: specific whole-genome duplication; CCT: cucurbit-common tetraploidization

To infer the chromosome evolution of wax gourd (*n* = 12) and other cucurbits, including cucumber (*n* = 7)^1^, melon (*n* = 12)^2^, watermelon (*n* = 11)^3^, bottle gourd (*n* = 11)^4^, and pumpkin (*n* = 20)^21^, we identified syntenic blocks across their genomes, using wax gourd as the reference (Fig. 2a). Analysis of the syntenic relationships shows that the integrity of six wax gourd chromosomes (chromosomes 2, 3, 5, 7, 8, and 10) was essentially preserved in pumpkin, from tribe *Cucurbiteae*, as well as in other species from tribe *Benincaseae*. The 12 melon chromosomes were previously proposed as the most ancestral karyotype from the then available cucurbit genomes^4^; however, only five melon chromosomes (chromosomes 2, 8, 9, 10, and 12) were well preserved in the pumpkin genome (Supplementary Fig. 7).

Next, we analyzed the syntenic relationships of the wax gourd and melon genomes vs. the bottle gourd genome, which has the most preserved ancestral karyotype among cucurbits after melon. Four wax gourd chromosomes (chromosomes 2, 3, 10, and 12) show nearly one-to-one syntenic relationships with bottle gourd chromosomes, whereas only two (chromosomes 2 and 8) show such a relationship in melon (Supplementary Fig. 8). Given the lowest evolutionary rate of wax gourd among the sequenced cucurbits, these data support the hypothesis that wax gourd represents the most ancestral genome among these species.

The ancestral chromosomes were inferred on the basis of syntenic relationships among cucurbit genomes, using the wax gourd as reference (Fig. 2a). The six wax gourd chromosomes (Bhi 2, 3, 5, 7, 8, and 10), showing a one-to-one relationship with the pumpkin genome, were considered as proto-chromosomes before the cucurbit-common WGD. Despite the integrity of wax gourd chromosome 4 (Bhi 4) is not being preserved in pumpkin, it has a one-to-one syntenic relationship with melon chromosome 3, indicating that it is also a proto-chromosome. The seven wax gourd chromosomes (Bhi 2, 3, 4, 5, 7, 8, and 10) were ordinally named proto-chromosomes 1–7. In addition, large patches of chromosome segments shared by extant genomes can be used to infer other proto-chromosomes. For example, wax gourd chromosome Bhi 9 could be found to occur in partite manner in other genomes, and each part is independent of the other one, and at the mean time independent of other chromosomes; this leads to the definition of proto-chromosomes 8 and 9. Similarly, we inferred proto-chromosomes 11, 12, 14, and 15. Some large patches have linked co-existence in extant genomes, showing that they could have originated from the same proto-chromosome. For example, one patch in Bhi 1 and another in Bhi 11 co-occurs in four genomes, especially two times in Cma, showing that they should be from the same proto-chromosome; this leads to the inference of proto-chromosome 13. Similarly, we inferred proto-chromosome 10. These data suggest that the ancestral genome of these sequenced cucurbit genomes has 15 proto-chromosomes.

The evolution of chromosomes in other sequenced cucurbits was investigated assuming the 15 ancestral chromosomes to have served as their origin (Fig. 2b). After wax gourd, the melon genome best preserved the ancestral karyotype of cucurbits, as previously reported^4^, with seven melon chromosomes (chromosomes 2, 3, 6, 8, 9, 10, and 12) derived directly from the ancestral ones. Despite the recent WGD in the pumpkin genome, 6 (chromosomes 3, 7, 10, 13, 19, and 20) of the 20 chromosomes remained in the ancestral state. The bottle gourd genome retained three ancestral chromosomes (chromosomes 1, 6, and 10), whereas all chromosomes of cucumber and watermelon were formed through a number of fusions and fissions. This information will be fundamental for comparative genomics in cucurbits.

### Repeat expansion leads to large genome size in cucurbits

The number of protein-coding genes and highly conserved syntenic blocks in the wax gourd genome are comparable with those in the genomes of other sequenced species in the tribe *Benincaseae*, including cucumber, melon, and watermelon (Supplementary Fig. 9, Supplementary Table 7). However, the assembled genome size of wax gourd (913.0 Mb) is at least twofold larger than that of the other three species (200.0–400.0 Mb). The absence of a recent WGD event in wax gourd suggests that this large genome size did not result from a specific WGD event (Fig. 1b).

**Fig. 3:**
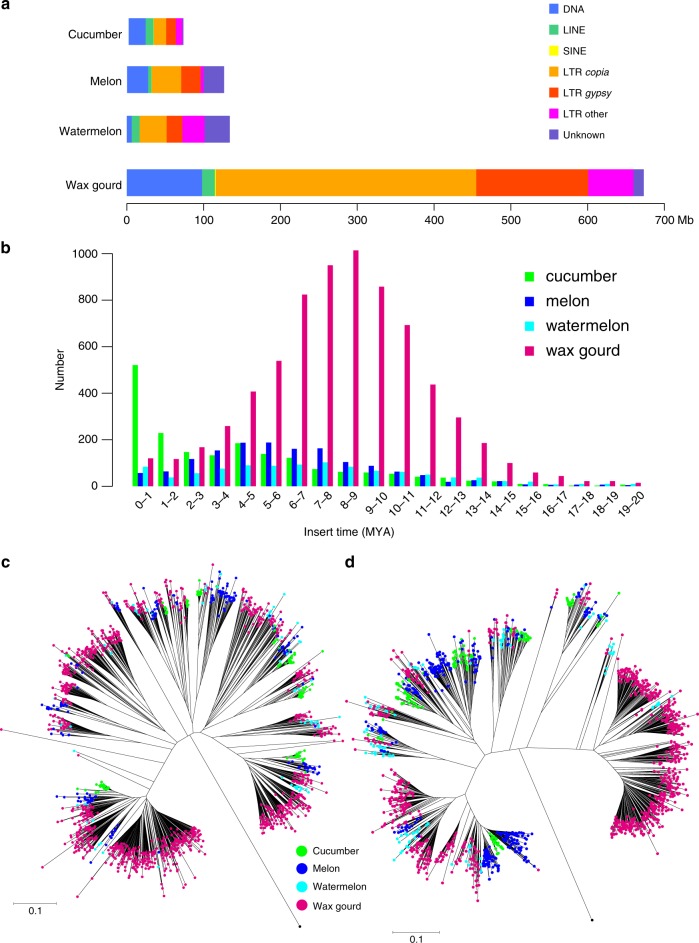
Expansion of repeats in wax gourd genome. **a** Transposable elements content in cucumber, melon, watermelon, and wax gourd genomes. **b** Distribution of insertion times for LTR retrotransposons in cucumber, melon, watermelon, and wax gourd. Phylogenetic relationships of *Copia* (**c**) and *Gypsy* (**d**) retrotransposons across cucumber, melon, watermelon, and wax gourd. Source data are provided as a Source Data file

Comparing the content of various repeats in the four species indicates that the length of DNA transposons and long terminal repeat (LTR) retrotransposons, including *Copia*, *Gypsy*, and other elements, in wax gourd is much greater than that in the other three species (Fig. 3a). For example, the length of *Copia* elements in wax gourd is ~20-fold longer than that in cucumber and nine-fold longer than that in melon and watermelon (Supplementary Table 8). Therefore, the substantial accumulation of transposable elements (TEs) and especially LTR retrotransposons contributes greatly to the large genome size of wax gourd.

To trace the history of the greatly expanded repetitive sequences in wax gourd, we estimated insertion times and analyzed the phylogenetic relationships of LTR retrotransposons, the most abundant repeats, using the 7136 full-length LTRs predicted in the wax gourd genome. LTRs accumulated gradually in the wax gourd genome before the divergence (~16.3 MYA) of wax gourd and watermelon, and peaked at around 9 MYA after speciation (Fig. 3b). LTRs accumulated earlier and faster in the wax gourd genome than in the three other *Benincaseae* species (Fig. 3b).

The recent substantial proliferation of LTRs in cucumber is not observed in wax gourd. In addition, we inferred phylogenies for the reverse transcriptase (RT) domain of both *Copia* and *Gypsy* elements (Fig. 3c, d). A number of diverse and ancient LTR subfamilies are present in all four species, along with numerous species-specific LTRs, especially in the wax gourd genome. Most LTRs were greatly expanded in wax gourd after speciation, and this ancient species-specific process led to the large extant genome of wax gourd.

### Genomic variations and population structure of wax gourd

To explore genetic variations in the wax gourd germplasm, 146 wax gourd accessions, including 13 wild accessions, 16 landraces, and 117 cultivated accessions, were selected and re-sequenced using Illumina sequencing technology (Supplementary Fig. 10). We generated 2.9 Tb of high-quality, cleaned sequences with an average ~15.68-fold and 95.38% coverage rate of the wax gourd genome (Supplementary Data 2). Mapping the reads onto the wax gourd genome identified a final set of 16,183,153 high-quality single-nucleotide polymorphisms (SNPs) (Supplementary Data 3) and 2,190,214 small insertions and deletions (InDels). Among the SNPs, 170,365 are missense SNPs, 2047 are nonsense and 1258 are located at splice site acceptors or donors. This variation data set represents a new resource for wax gourd biology and genetic breeding.

**Fig. 4:**
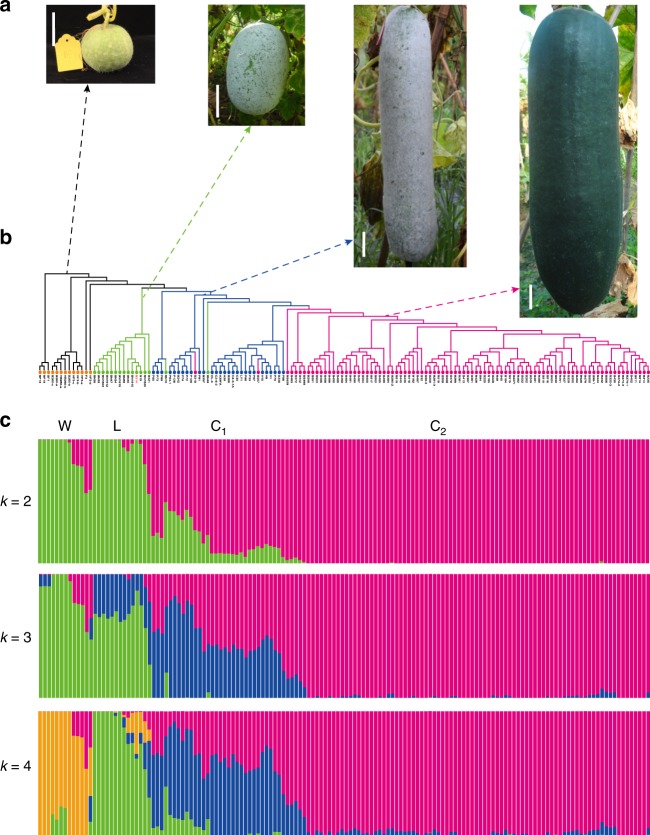
Population structure of 146 wax gourd accessions. **a** Fruit morphology of the four groups. Bar is 5 cm. **b** Neighbor-joining phylogenetic tree constructed using SNPs at fourfold-degenerate sites. Wild group (W) contains wide accessions from India, Japan, and China (orange); accessions of Landrace group (L) are mainly form Xishuangbanna region of China (green). Two cultivated groups contain accessions bearing large fruit, with group C1 (blue) being covered by wax, but not in group C2 (magenta). **c** Model-based clustering analysis with different cluster numbers (*k* = 2, 3, and 4). The *y*-axis quantifies cluster membership, and the *x*-axis lists the different accessions. The orders and positions of these accessions on the *x*-axis are consistent with those in the neighbor-joining tree. The source data underlying **b** and **c** are provided as a Source Data file

To infer the population structure of wax gourd germplasm, phylogenetic, Bayesian clustering and principal component (PCA) analyses were performed using fourfold-degenerate sites (Fig. 4, Supplementary Figs. 11 and 12). All results support clustering of wax gourd accessions into four groups. The 13 wild accessions form a wild group (W) clade with some admixtures. Of the 16 landraces, 14 group together as a landrace group (L), with some admixtures from wild and cultivated accessions. The remaining 117 cultivated and two landrace accessions belong to the cultivated group and could be divided into two distinct sub-groups, one with fruit wax (sub-group C1) and the other without (C2).

The phylogenetic tree suggests that cultivated wax gourds were most likely formed through a two-step evolutionary process, including domestication from wild to landrace accessions and improvement from landrace to cultivated accessions (sub-groups C1 and C2). Sub-group C2 without fruit wax should have been bred from sub-group C1 with fruit wax. As in other cucurbits^22^, the genome-wide nucleotide diversity (*π*) of the wild group (5.9 × 10^−3^) is far higher than that of the landrace (1.1 × 10^−3^) and cultivated groups (0.4 × 10^−3^), indicating a highly diverse gene pool in the wild group, which could be a valuable genetic resource for wax gourd improvement.

### Candidate regions/genes conferring fruit size under selection

For many crops, an essential change during the process of domestication and improvement was the increase of fruit and/or seed size. The fruit mass of wax gourd was increased from ~0.5 kg in the wild accessions to ~2.0 kg in the landrace accessions, during domestication, and subsequently from ~2.0 kg in the landrace accessions to ~10 kg in the cultivated accessions during improvement. Histological and morphological analyses of the fruit between landrace accession B214 with fruit of ~2.0 kg and cultivated accession B227 with fruit of ~20 kg indicate that increased fruit size is determined by both number and volume of cells (Supplementary Fig. 13).

**Fig. 5:**
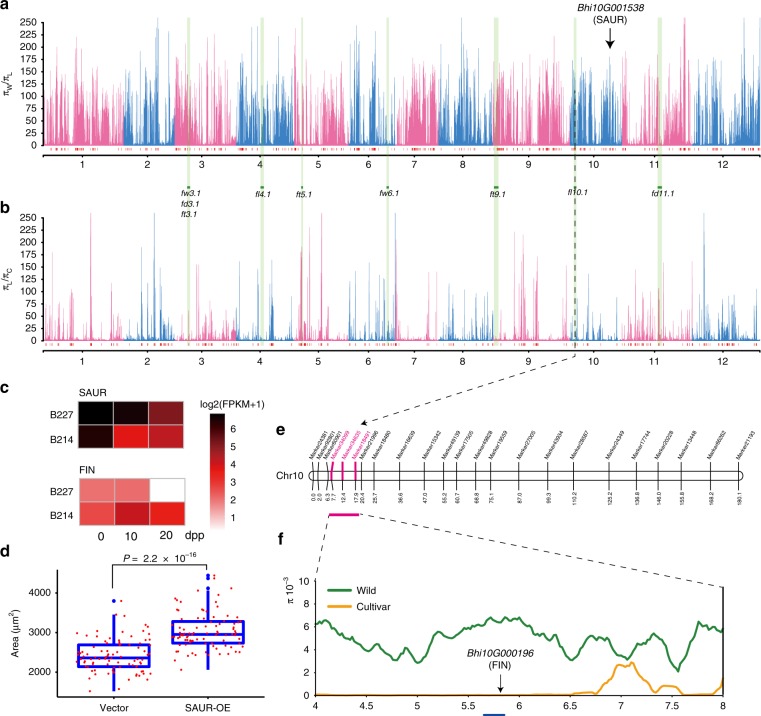
Domestication/improvement selective sweeps and identification of fruit-size candidate genes in wax gourd. **a**, **b** Genome-wide distribution of selective sweeps during domestication and improvement. Red vertical boxes illustrate selective sweeps and light green bars illustrate QTLs related fruit size. **c** Differential expression of candidate genes related to fruit size between large (B227) and small (B214) fruited accessions, at three developmental stages. **d** Boxplots show the epidermal pavement cell size of wax gourd cotyledon with SAUR overexpression and empty vector (OE: overexpression), *p* value was calculated using one-sided Student’s *t* test. **e** A major fruit length QTL on chromosome 10. **f** Domestication sweeps within the genetically mapped QTL interval. Horizontal blue lines indicate sweep regions. The source data underlying  **a**, **b**, and **d** are provided as a Source Data file

To identify potential selective signals related to fruit size and other important agronomic traits, during wax gourd domestication (wild vs. landrace) and improvement (landrace vs. cultivated), we scanned genomic regions with a drastic reduction of nucleotide diversity (top 10%). The sweep regions were further filtered using top 50% XP-CLR scores^23^. Finally, we detected 234 domestication sweeps ranging from 200 to 2580 kb in length (456 kb on average) (Fig. 5a, Supplementary Data 4 and 5) and 168 improvement sweeps (Fig. 5b, Supplementary Data 6 and 7) ranging from 200 to 2440 kb in length (475 kb on average). The domestication and improvement sweeps occupy 106.7 Mb (11.6% of the assembly genome), including 3939 genes, and 80.3 Mb (9.1%), including 2251 genes, respectively.

Of note, on chromosome 10, a domestication sweep region from 47.84 to 48.34 Mb contains 21 genes. Among these genes, *Bhi10G001538* encoding a small auxin-up-regulated RNA (SAUR) is significantly up-regulated in the large-fruited accession (B227), at three developmental stages, compared to that in the small-fruited one, and is highly expressed specifically in the fruit (Fig. 5c). In cucumber, its ortholog *Csa2G258100* is also significantly up-regulated in long-fruited line^24^ (Supplementary Fig. 14), and was mapped in the quantitative trait locus (QTL) interval *fd2.1* for fruit diameter^25^. Moreover, SAUR was reported to be involved in regulating plant growth and development by promoting cell expansion^26^. To confirm the function of *Bhi10G001538* gene in cell expansion in wax gourd, we transiently expressed *35S-MYC*-*Bhi10G001538*, and a vector control, in cotyledons by agroinfiltration. These assays indicated that the cell size of cotyledonary epidermal pavement cells, at 5 days post infiltration (dpi), was significantly larger than that in cotyledons expressing a vector control (Fig. 5d). Therefore, *Bhi10G001538* may be an important candidate domestication gene conferring large fruit size in wax gourd.

To further explore for genes potentially related to fruit size, during domestication and improvement, we performed QTL mapping of fruit-size-associated traits, using a segregating population^27^ derived from a cross between landrace accession B214, which should be an admixture of wild, landrace, and cultivated wax gourds (Fig. 4c), and a cultivated accession, B227. We mapped nine QTLs, including two for fruit mass (*fw3.1* and *fw6.1*), two for fruit length (*fl4.1* and *fl10.1*), two for fruit diameter (*fd3.1* and *fd11.1*), and three for thickness of fruit flesh (*ft3.1*, *ft5.1*, and *ft9.1*) (Fig. 5, Supplementary Data 8). Among these nine QTLs, six (*fw3.1*, *fd3.1*, *ft3.1*, *ft5.1, fl10.1*, and *fd11.1*) contain both domestication and improvement sweeps, with the selection occurring at different positions; these regions may harbor multiple genes related to fruit size. For instance, within the physical interval of the *fl10.1* QTL for fruit length, one domestication sweep from 5.20 to 6.56 Mb contains 55 genes (Fig. 5e, f). Among these genes, *Bhi10G000196* is the homolog of *SlFIN* (*Solyc11g064850*) in tomato, the mutation of which can cause enlarged tomato fruit^28^. Moreover, *Bhi10G000196* is significantly down-regulated in the large-fruited accession, at three developmental stages, compared to that in the small-fruited accession (Fig. 5c) and highly expressed in the fruit. These data support a role for this gene in the increase of fruit size during wax gourd domestication.

Genome-wide association studies (GWASs) were also performed for traits related to fruit size, including fruit weight, length, diameter, and thickness of fruit flesh, using the 146 accessions sequenced. Here, we identified 30 significant association signals at a threshold of −log_10_(*p*) = 6 (Supplementary Fig. 15 and Data 9). Among them, 11 overlapped with domestication/improvement sweeps containing 36 genes. Thus, these 11 genes also provide candidate domestication genes responsible for large fruit size.

## Discussion

In this study, we present a high-quality draft genome sequence for wax gourd, which has a larger genome size than other cucurbit species, such as cucumber, melon, and watermelon. Comparative analysis of the available whole genomes of cucurbit species provides evidence in support of the model in which the wax gourd genome is the most ancestral karyotype of the cucurbit species investigated. Wax gourd belongs to a monotypic genus in which no new speciation occurred. In mammals, taxa with low rates of speciation are associated with the so-called cold genomes, which have lower TE activity^29^. Considering that the large proportion of repetitive sequences in wax gourd are ancient TEs with low activity and TE activity is associated with speciation^30,31^, we speculate that the large number of ancient repeats in wax gourd, which has the slowest evolutionary rate among cucurbits, might be related to the ancestral karyotype and the nature of the monotypic genus.

All analyzed species here are from the tribe *Benincaseae* and *Cucurbiteae*. There are a total of 15 tribes in the Cucurbitaceae family. With the availability of more genome sequences of cucurbit species from other tribes, the accurate ancestral genome and the evolutionary scenario of cucurbit genomes will be investigated comprehensively. It seems that the species with 12 chromosomes such as wax gourd and melon were preferred to preserve the ancestral chromosomes. Chayote (*Sechium edule*) is a popular fruit plant from the tribe *Sicyeae*, and it also has 12 chromosomes. Thus, the genome of chayote may provide insights into the genome evolution of cucurbits.

Most wax gourd cultivars bear a giant fruit, but its wild form has a small fruit. How wax gourd domesticated from wild to cultivated one remains largely unknown. By resequencing 146 accessions, we generated a genomic variation map for wax gourd and revealed its population structure and the genetic basis of diversity. A number of putative genome regions under domestication or improvement were identified. Several candidate genes were proposed to be involved in the process from wild small fruit to cultivated large one. Interestingly, homologs in other species of *Bhi10G001538* and *Bhi10G000196* are also probably responsible for fruit size.

In summary, the draft genome sequence and genomic variation map of wax gourd provide insights into the genome evolution of cucurbit species and the genomic basis of wax gourd’s diversity. Moreover, these data and information are valuable resources for wax gourd research and breeding, and for comparative genomic analysis of cucurbit species.

## Methods

### Sequencing and assembly of the wax gourd genome

For genome sequencing, genomic DNA was extracted from leaf buds of wax gourd inbred line B227 using a modified cetyltrimethylammonium bromide (CTAB)^32^ protocol. Three paired-end (with insert sizes of 180 and 500 bp) and five mate-pair sequencing DNA libraries (with insert sizes of 2 and 8 kb) were constructed following the Illumina standard protocol (Supplementary Table 1). A total of 29.3 Gb paired-end and 12.1 Gb mate-pair sequences was generated on sequencing platform Illumina Hiseq 2000. All low-quality reads were filtered. In addition, ~15 G subreads were generated using SMRT sequencing technology on the PacBio RSII platform. The average length of the subreads was 6.1 kb.

The Illumina paired-end reads were assembled into contigs using the ALLPATHS-LG-44837 software^33^. These contigs were then connected into scaffolds based on the mate-pair reads. The gaps in the preliminary assembly were filled by integrating PacBio subreads with PBjelly^34^. Furthermore, we used the ALLMAPS^35^ software to anchor those scaffolds onto 12 pseudo-chromosomes, based on a published high-density genetic map^10^.

### Genome annotation

RepeatModeler (v1.0.4) was used for de novo prediction of TEs in the wax gourd genome. These de novo predicted repeats, together with the TIGR plant repeats database (http://plantrepeats.plantbiology.msu.edu), were used to mask the repeats in the wax gourd genome. These masked repeats were then classified into different types based on the annotation of RepeatMasker.

Evidence from transcript mapping, ab initio gene prediction, and homologous gene alignment was combined to predict protein-coding genes in the repeat-masked wax gourd genome. RNA-seq data from five tissues were aligned against the wax gourd genome, using HISAT2^36^, and were assembled using stringtie (1.2.2)^36^. The assembled transcripts were further processed by PASA (v2.0.2)^37^, and then were used for ab initio prediction as well as the evidence of transcript mapping. SNAP (2006-07-28)^38^, GlimmerHMM (v2.0.4)^39^, and AUGUSTUS (v3.1)^40^ were used for ab initio gene prediction. For homologous gene alignment, non-redundant plant protein sequences, downloaded from Uniprot (http://www.uniprot.org), were aligned to the genome using Wise (2.4.1)^41^. Finally, gene structures were predicted on the basis of a weighted consensus of all the evidence using EVM^42^.

All predicted proteins were aligned to GenBank NR, the *Arabidopsis* protein, UniProt (Swiss-Prot), and InterPro databases using BLAST or interproscan (5.16–55.0)^43^ for functional annotation of protein-coding genes. Based on alignment results, the GO and Uniprot annotations were assigned for each protein.

### Gene families and phylogenetic analysis

We used the OrthoMCL package (version 2.0.9)^44^ to identify gene families/clusters between the wax gourd and 11 other plant species, including six other cucurbits (cucumber, melon, watermelon, bottle gourd, pumpkin, bitter gourd), three rosid species (soybean, *Arabidopsis*, grape), one asteroid (tomato), and one monocot (rice). We investigated the dynamic evolution of gene families using Cafe ´software (version 3.1)^45^ with a probabilistic graphical model. Phylogenetic relationship among these 12 plant species was resolved using the RAxML package (version 8.1.13)^46^, based on the 463 high-quality single-copy orthologous genes. Divergence times were estimated by the program MCMCtree in PAML (version 3.15) (http://abacus.gene.ucl.ac.uk/software/paml.html), based on known divergence time between cucumber and melon (about 10 MYA)^47^.

### Gene collinearity and Ks analysis

Protein sequences within a genome or between different genomes were aligned by BLASTP. Matched genes with *e* value <1e − 5 were considered as potential homologous genes. Next, syntenic blocks within a genome or between different genomes were determined based on the detected homologous gene pairs using ColinearScan^48^. WGD events were inferred from the syntenic relationships within a genome.

Synonymous nucleotide substitutions on synonymous sites (Ks) were estimated using the Nei–Gojobori approach^49^ implemented in the Bioperl Statistical module. We used normal distribution to represent the complex Ks distribution, and the principle one was used to represent the corresponding evolutionary event^20,50^. To infer the evolutionary events and rates properly, we corrected the Ks on the basis of ECH and CCT WGD events. First, the inferred Ks peak from ECH-produced duplicated genes was aligned to have the same value with that of grape, which has been evolved the slowest. Then, all related cucurbits have the same distribution peak at the CCT event with that of wax gourd, which has the slowest evolutionary rate among these cucurbits.

### Evolutionary scenario of cucurbit genomes

By comparing different cucurbit genomes, phylogenetically, we adopted a bottom-up approach to reconstruct the ancestral cell karyotypes of cucurbit plants. First, by inferring putative homologous genes and collinear genes, we drew homologous gene dot plots within a genome and between genomes. Ks values were estimated to infer collinear genes produced by different events, and the information was integrated into the dot plots. Second, since pumpkin is the outgroup of other studied cucurbits, we checked the dot plots to assess whether its chromosomes or main structures of its chromosomes were shared by other cucurbit plants. Third, the fusion and fission events during genome evolution of cucurbit species from their ancestral chromosomes were determined.

### Analysis of full-length LTR retrotransposons

We used LTR_Finder (v1.0.6)^51^ to de novo detect full-length LTR retrotransposons in four species of Cucurbitaceae (cucumber, melon, watermelon, and wax gourd) genomes, with the following command line ltr_finder genome.fa -s tRNAdb/Athal-tRNAs.fa -a ps_scan > result.txt. Next, we obtained candidate full-length LTRs by filtering those that overlapped. These full-length LTR retrotransposons were then translated into amino acids, in six frames, with the best being selected. Their functional domains were predicted using the software HMMER, based on the Pfam database. Paralogs of the RT domain, specific for the *Copia* and *Gypsy* super-families, were then detected based on the functional domains and their orders.

The RT protein sequences were aligned using MUSCLE (v3.8.31)^52^ and neighbor-joining (NJ) trees were built using MEGA (default parameters) for the *Copia* and *Gypsy* super-families. The two ends of these LTR retrotransposons were aligned with MUSCLE, and the nucleotide distance (*D*) was estimated using the Kimura two-parameter (K2p) (transition–transversion ratio) criterion, as implemented in the distmat program in the EMBOSS package (v6.6.0)^53^. Then rate of nucleotide substitution (*μ*) were inferred as following strategy^54^: first, detecting LTR-NRR (not repeat-related flanking sequence) orthologous insert sequences between cucumber and melon; second, estimating nucleotide distance (*D*) of orthologous LTRs between cucumber and melon; finally, substitution rates (*μ*) were inferred using the formula: *μ* = *D*/2*T* based on the known divergence time between cucumber and melon^47^. The insertion time (*T*) of an LTR retrotransposon was calculated using Eq. 1:

1 $${T} = {D}/2{\upmu},$$

where *μ* is 4.5e^−9^.

### Genome variation map of wax gourd

A total of 146 wax gourd accessions were included in this study, all being collected by Guangdong Academy of Agricultural Sciences. Genomic DNA was extracted from leaf buds using the CTAB method^32^. Paired-end Illumina genomic libraries with insert sizes of 300–500 bp were prepared and sequenced on an Illumina Hiseq4000 platform (Illumina Inc., USA) with the read length of 150 bp.

The paired-end sequence reads from each accession were mapped onto the wax gourd reference genome, using BWA (version: 0.7.17-r1188)^55^ with the default parameters. SAMtools (version: 1.6-3-g200708f)^56^ was used to convert mapping results into the BAM format, to sort mapping results according to mapping coordinates and to remove PCR duplicated reads. The resultant files were then used in the following procedures for variant detection.

Variant calling was performed using the SNP detection procedure of the Genome Analysis Toolkit (version: v3.2-2-gec30cee)^57^. These SNPs were further filtered using the following criteria: (i) one position with more than two alleles was considered to be a polymorphic site in the population; (ii) total sequencing depth had to be >150 and <6570, the sequences with the depth >6570 were considered as repeat sequences; (iii) nearest SNPs had to be >1 bp away; and (iv) we filtered out sites at which <85% of the lines appeared to be homozygous and sites with a proportion of heterozygous genotypes greater than three times that of the homozygous genotypes with the minor allele. In addition, small InDels (≤5 bp in length) were also identified with the same criteria.

### Phylogenetic and population analysis

To build a NJ tree, we screened a subset of 6585 SNPs at fourfold-degenerate sites (minor allele frequency (MAF) >5% and missing data <10%) from the identified SNPs of the wax gourd accessions. These SNPs should be under lower selective pressure, thus they are considered to be more reliable in reflecting population structure and demography. We constructed a phylogenetic tree using MEGA (version 6)^58^ with 1000 bootstrap replicates.

Using the same SNP data set, we also investigated the population structure using STRUCTURE (version 2.3.4)^59^, on the basis of allele frequencies. To determine the most likely group number, STRUCTURE was run 20 times on 1000 randomly selected SNPs at fourfold-degenerate sites for each *K* value from 2 to 20. After determining Δ*K*, we used 6585 SNPs at fourfold-degenerate sites to determine the group membership of each accession by 10,000 iterations, with *K* values from 2 to 4. In addition, we performed PCA^60^ using the same data set. Two-dimensional coordinates were plotted for the 146 wax gourd accessions.

### Identification of domestication and improvement sweeps

Nucleotide diversity (*π*) and Tajima’s *D*^61^ in wild (W), landrace (L), and cultivated (W) wax gourd groups were calculated on the basis of the genotypes of each accession using BioPerl. A sliding window approach was used to calculate *π* in the wax gourd genome with a window size of 200 kb and a step size of 20 kb. By scanning the ratios of genetic diversity between W and L (*π*_W_/*π*_L_ domestication step), as well as between L and C (*π*_L_/*π*_C_ improvement step), we selected windows with the top 10% of ratios (97.3 and 20.9 for domestication and improvement, respectively) as candidate domestication/improvement regions. To improve the predictive accuracy, only candidate selective sweeps having the top 50% of XP-CLR scores were kept. Finally, windows that were ≤200 kb apart were merged into a single region under selection.

### Measurement and statistics of cell size

To measure cell size in the wax gourd fruit of B227 and B214, 1-cm-thick sliced samples were cut from the outer, middle, and inner pericarp at different developmental timepoints, at 0, 5, 10, 15, 20, and 25 days after pollination (DAP). These sections were fixed in a solution of ethanol (70%), acetic acid, and formaldehyde (90:5:5 by volume) and then embedded into paraffin. Subsequently, 8-μm-thick-microtome sections were prepared (from cross and longitudinal, stained with hematoxylin–eosin), and examined and images collected by light microscopy. Cell size in each section was calculated by the ImageJ software; the top 30 cells in size were counted, and the mean and variance in cell size was calculated, for each development period examined. Measurements were made at three different sites of each tissue, for three sections from each fruit.

### Analysis of differentially expressed genes

The high-quality RNA was separately extracted from large (B227) and small (B214) fruited accessions at three developmental stages (0, 10, and 20 DAP), with three biological replicates (Supplementary Data 10). Libraries were constructed according to the protocol for the Illumina HiSeq4000 platform. All clean RNA-seq reads, generated from each sample, were mapped onto the assembly sequences, using TopHat2^36^ (version 2.1.0) with default parameters. The generated BAM format alignments, together with the gene GTF annotation file, were then fed to htseq-count (version 0.11.2) to compute the read counts. Differentially expressed gene (DEG) analysis was performed using the edgeR package implemented in R^62^. DEGs were identified using a false discovery rate set at <0.05 and fold change >2 as cutoffs. The FPKM (fragments per kilobase of exon model per million reads mapped) values were computed using stringtie^36^ (v1.3.4d).

### Immunoblotting

The agrobacterium GV3101 cells harboring the pSuper-1300-35S-SAUR and vectors were inoculated in LB culture medium and cultured overnight at 28 °C. The agrobacterium culture was centrifuged and suspended with IM (inoculation medium: 10 mM MES (2-(*N*-morpholino)ethanesulfonic acid), 10 mM MgCl_2_, 500 µM AS (acetosyringone)). After 2 h incubation, at room temperature in darkness, the agrobacterium culture was centrifuged and suspended again with IM, and the final concentration of agrobacterium (measured by OD_600_) was adjusted to 0.3, 0.6, and 0.9. The agrobacterium inoculum was infiltrated into healthy and fully expanded cotyledons of 1-week-old wax gourd plants using a needleless syringe. Total protein was extracted at 3 dpi and mouse anti-MYC (ABmart, Lot number: 294166) antibody was used at 1:3000 concentration for immunoblots. The cell size of cotyledonary epidermal pavement cells was investigated, at 5 dpi, on free-hand sections. Cell size in each section was calculated by the ImageJ software.

### GWAS of fruit-size-related traits

Fruit-size-related traits, including weight, length, diameter, and thickness, were evaluated three times during the spring of 2014, 2015, and 2016, at the Guangdong Academy of Agricultural Sciences (Supplementary Data 11). Since the phenotypic data across years were found to be highly consistent, the average values for each trait were used for further analysis. GWAS were performed using Emmax^63^. A total of 2,237,614 SNPs, with a missing rate ≤10% and MAF ≥5% were used, and the average values of fruit-size-related traits were utilized for the association study. GWAS threshold was set using *N* (the effective number of independent SNPs, *p* = 1/*N*). The effective number of independent SNPs was calculated using Genetic type 1 Error Calculator (GEC) software^64^. Finally, the signals with *p* < 10^−6^ were considered as the significantly associated sites.

### Reporting summary

Further information on research design is available in the Nature Research Reporting Summary linked to this article.

## Supplementary information

Supplementary Information

Peer Review

Reporting Summary

Description of Additional Supplementary Files

Supplementary data 1-11

Source data

## Data Availability

Data supporting the findings of this work are available within the paper and its Supplementary Information files. A reporting summary for this Article is available as a Supplementary Information file. The datasets generated and analyzed during the current study are available from the corresponding author upon request. The wax gourd genome sequences have been deposited in GenBank of NCBI with BioProject ID PRJNA430006 [https://www.ncbi.nlm.nih.gov/bioproject/?term=PRJNA430006]. The raw sequenced reads from the 146 wax gourd accessions have been deposited in the Genome Sequence Archive of the BIG Data Center under accession number CRA001259 and in the sequence read archive (SRA) of NCBI under accession number SRP224893. The raw transcriptome sequences have been deposited in the Genome Sequence Archive of the BIG Data Center under accession number CRA001814 and in the SRA of NCBI under accession number SRP224600. The source data underlying Figs. 1a, 3, 4b, c, and 5a, b, d, as well as Supplementary Figs. 1, 4, 7, 8, and 11 are provided as a Source Data file.
