# Supplementary material for: The wax gourd genomes offer insights into the genetic diversity and ancestral cucurbit karyotype
Source: Nat Commun. 2019 Nov 14;10:5158. doi: 10.1038/s41467-019-13185-3 (PMC6856369; doi:10.1038/s41467-019-13185-3)
Supplement: Supplementary file 1 — Supplementary Information [file 41467_2019_13185_MOESM1_ESM.pdf]

The wax gourd genomes offer insights into the genetic diversity and ancestral cucurbit karyotype

Xie *et al.*

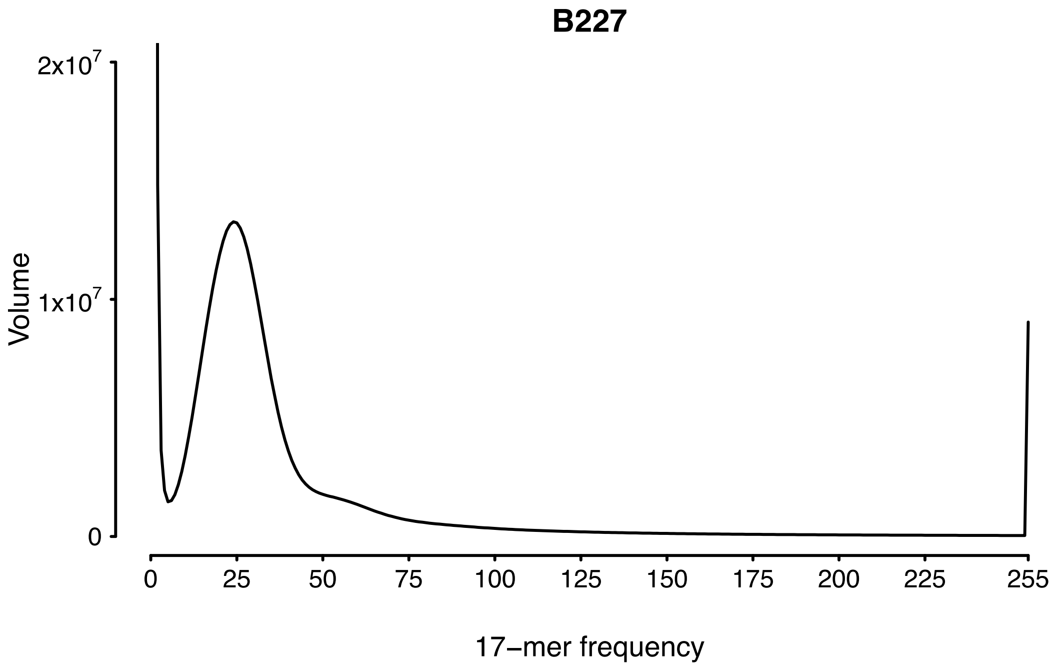

Supplementary Figure 1. Distribution of 17k-mer depth for the Illumina reads. Source data are provided as a Source Data file.

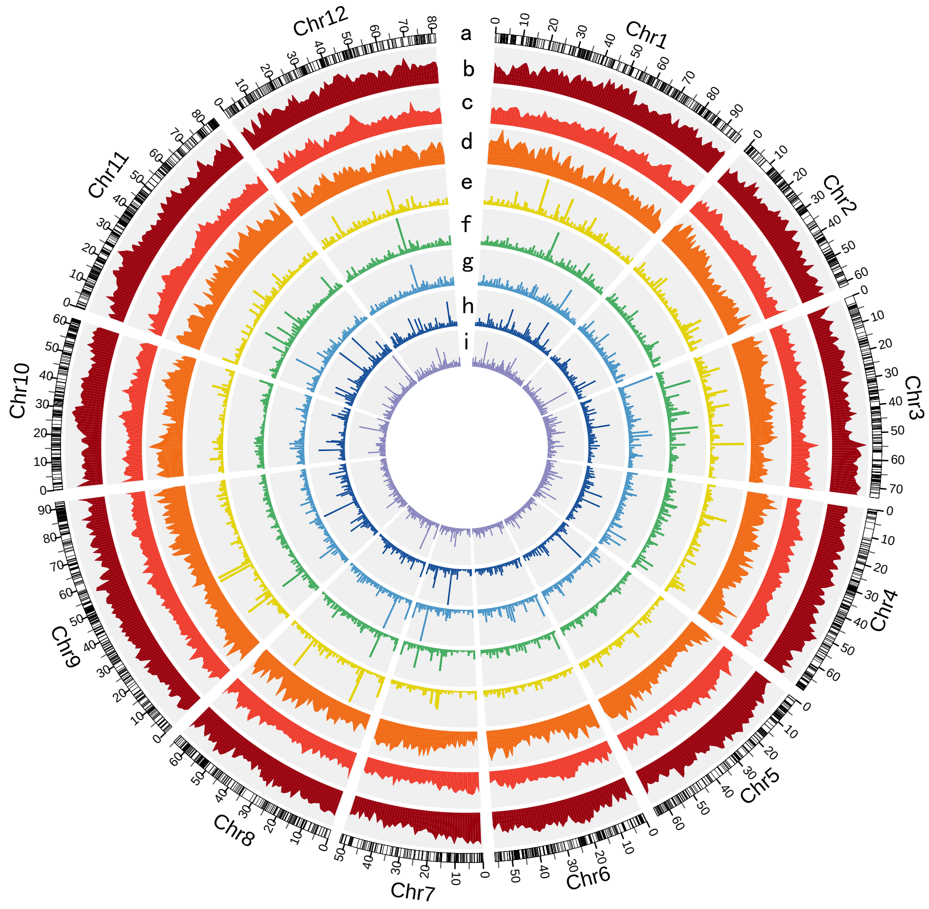

Supplementary Figure 2. Wax gourd genome. a Genetic markers distribution across 12 chromosomes. **b** Repeat coverage (50-90%). **c** GC content (30-40%). **d** Gene density (0-60). **e-i** Gene expression profile in root, stem, leaf, flower and fruit respectively (FPKM: 0-100). **b-i** are drawn in non-overlapping 1 Mb sliding windows.

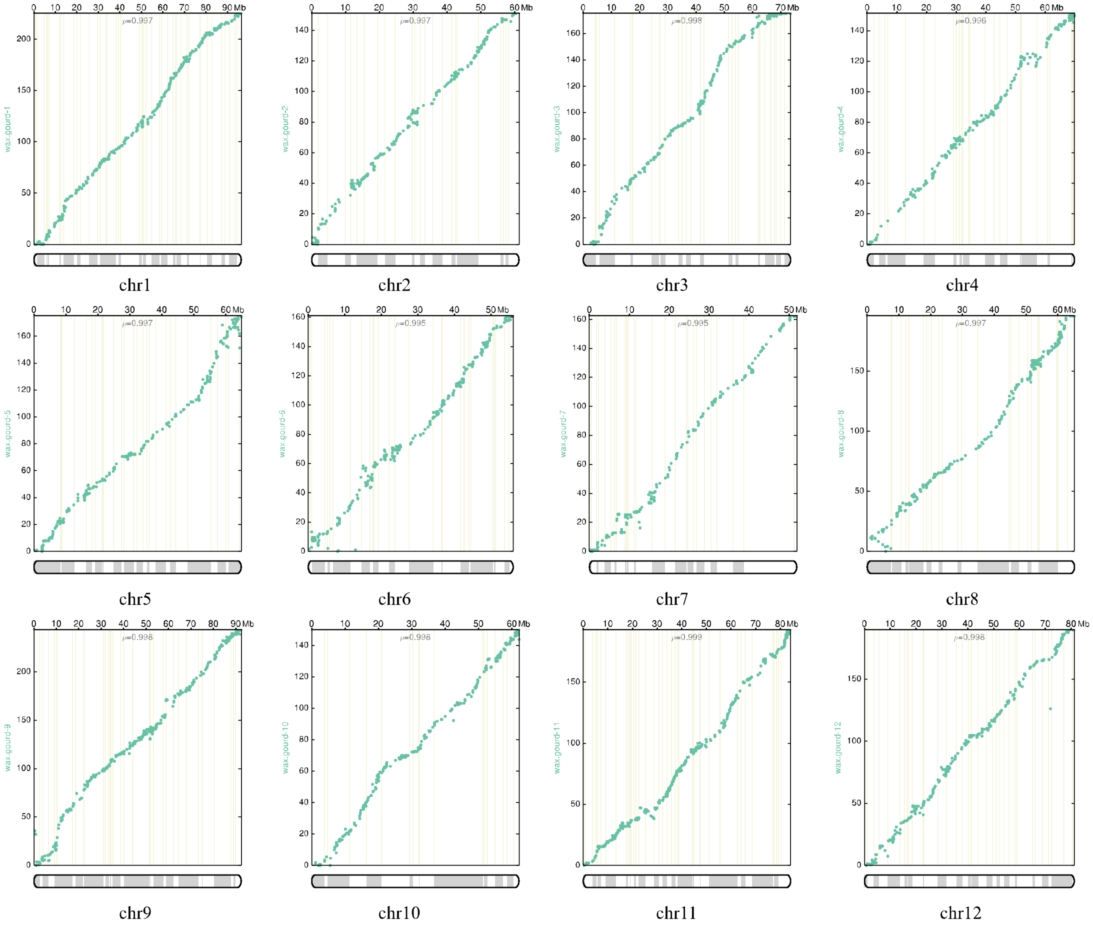

Supplementary Figure 3. Consistence of the wax gourd genome assembly with the high-density genetic map. The horizontal axes show the assembled scaffolds as boxes with grey white alternating shades on the pseudo-chromosomes. The vertical ones represent the genetic distance in the genetic map.

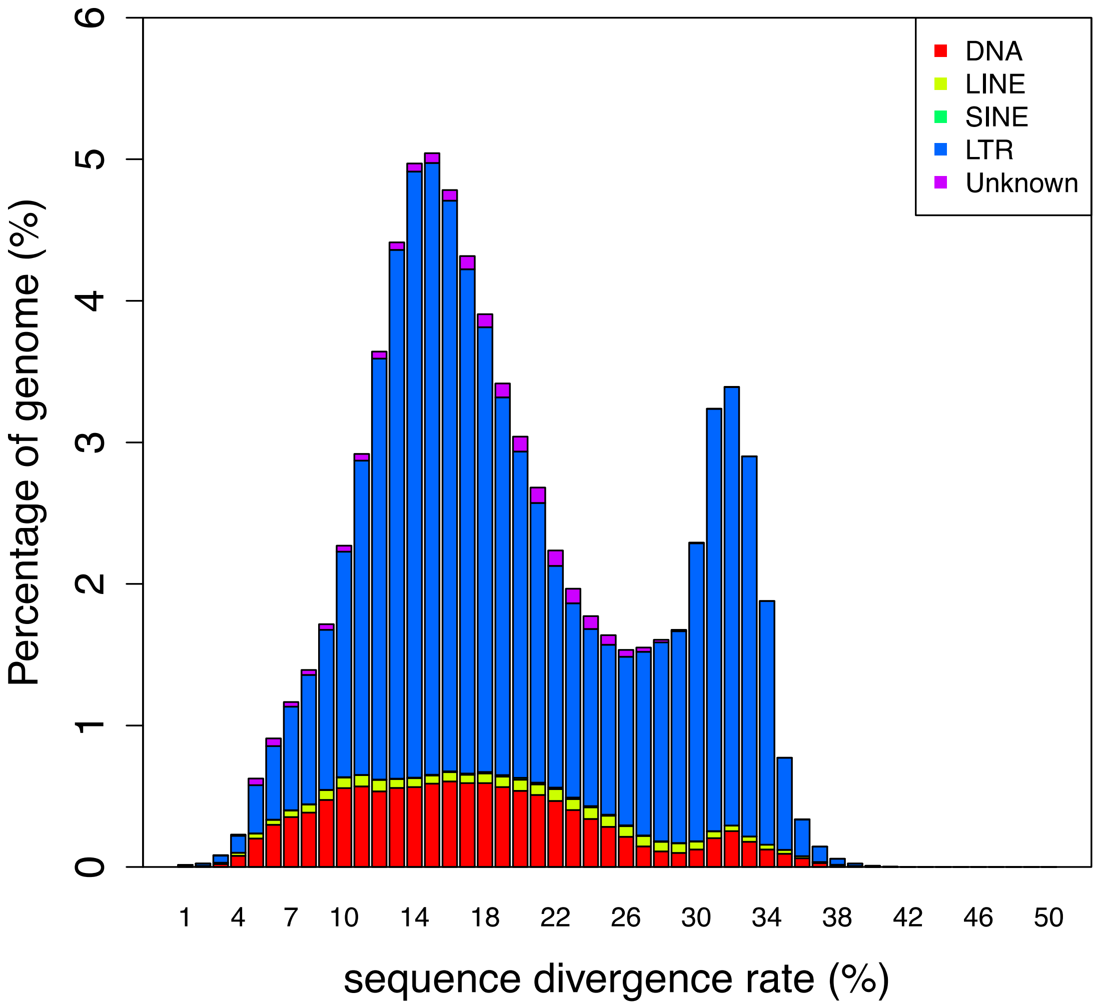

Supplementary Figure 4. The divergence rates of repeats in the wax gourd genome. Divergence rate: percentage of substitutions in the matching region compared with consensus sequence in the repeat library. Source data are provided as a Source Data file.

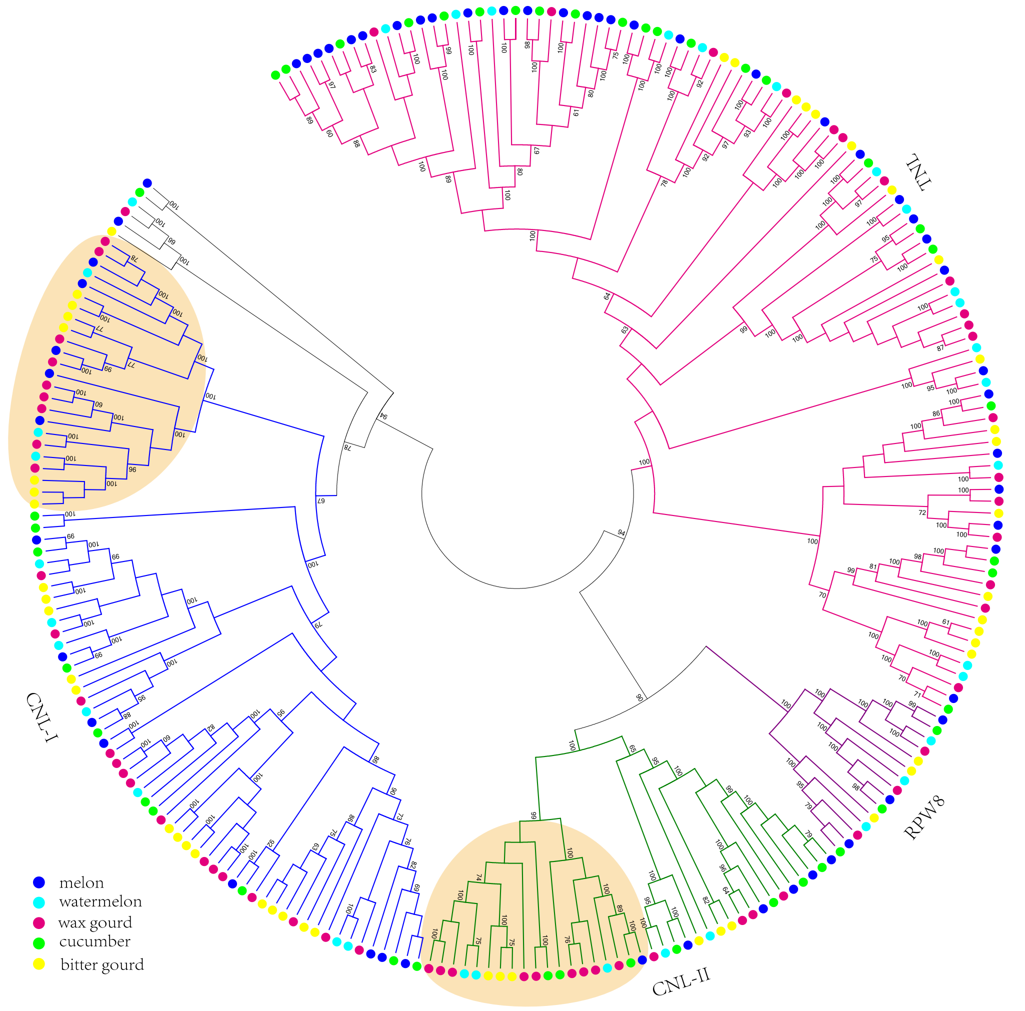

Supplementary Figure 5. **Phylogenetic tree of NBS R genes in five *Cucurbitaceae* species.** Cucumber, melon, watermelon, bitter gourd, and wax gourd were involved in the analyses. The tree was generated using protein sequences of the conserved NB-ARC domain.

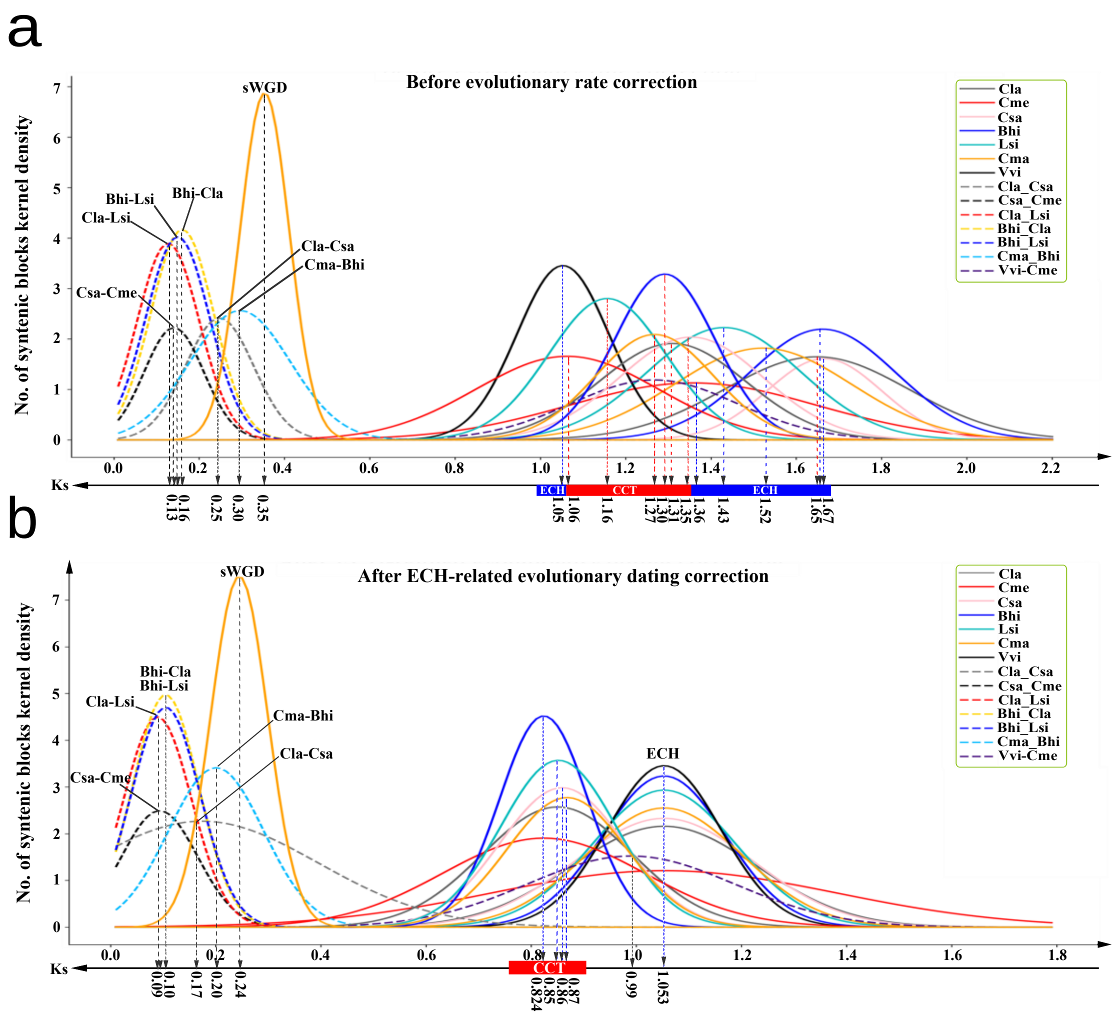

Supplementary Figure 6. Distribution of average synonymous substitution levels between collinear gene pairs in intergenomic blocks and intragenomic blocks. Ks: synonymous substitution. Solid curves: intergenomic blocks. Dashed curves: intragenomic blocks. **a** Before Ks correction. **b** After Eudicot-common hexaploidy (ECH) related evolutionary rate correction. Bhi: *Benincasa.hispida*, Cma: *Cucurbit maxima*, Cme: *Cucumis melo*, Lsi: *Lagenaria siceraria*, Cla: *Citrullus lanatus*, Csa: *Cucumis sativus*, Vvi: *Vitis vinifera*.

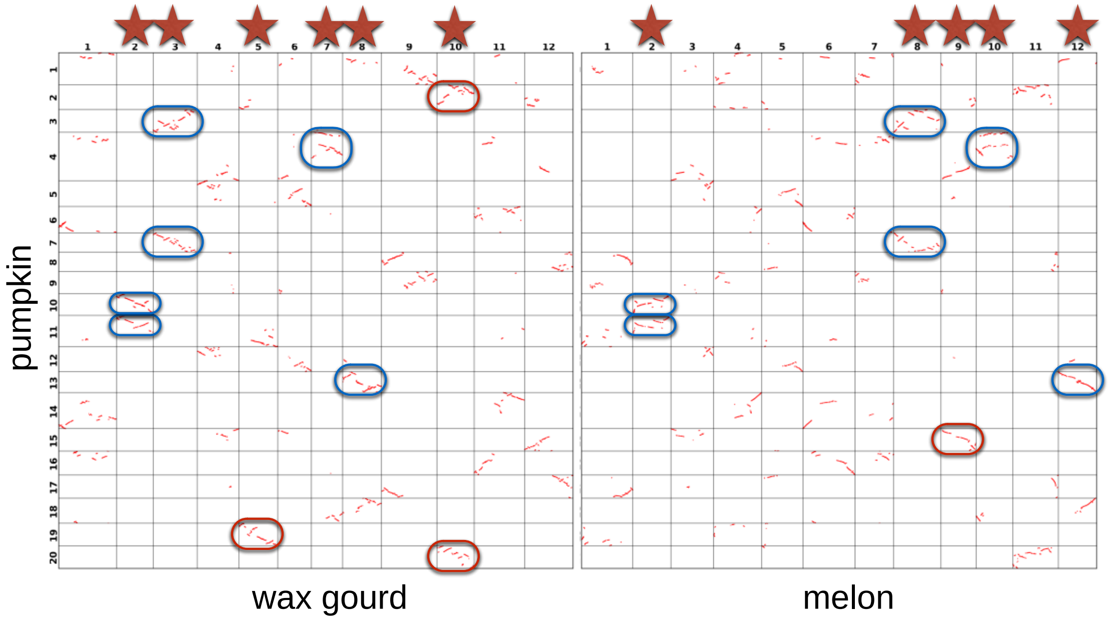

Supplementary Figure 7. The syntenic dotplots of wax gourd and melon versus pumpkin genome. The chromosomes marked by asterisk mean the whole chromosome have been well preserved in pumpkin genome. Source data are provided as a Source Data file.

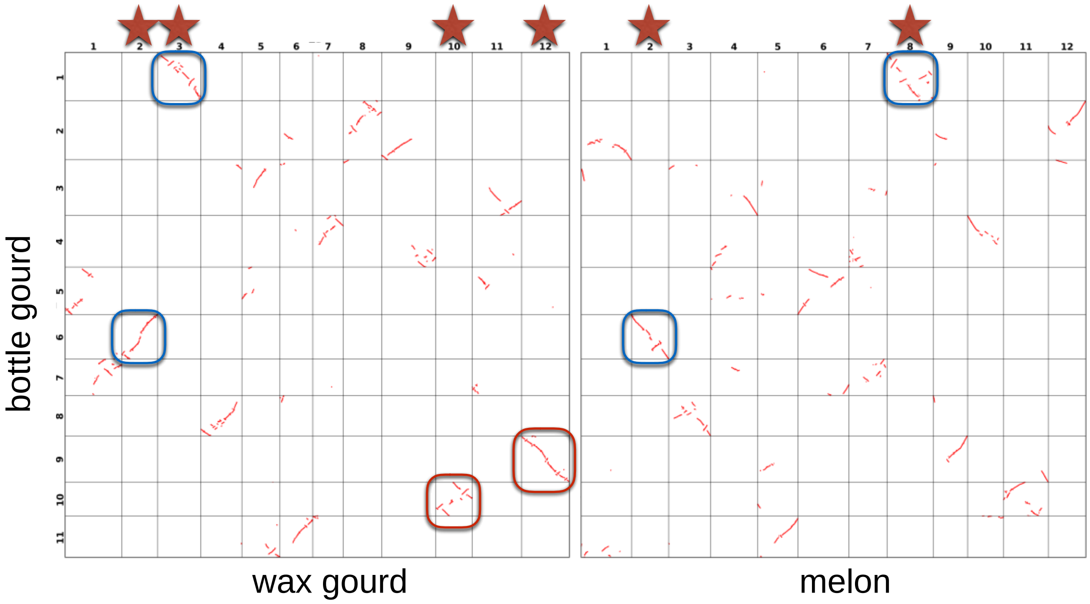

Supplementary Figure 8. The syntenic dotplot of wax gourd and melon versus **bottle gourd.** Source data are provided as a Source Data file.

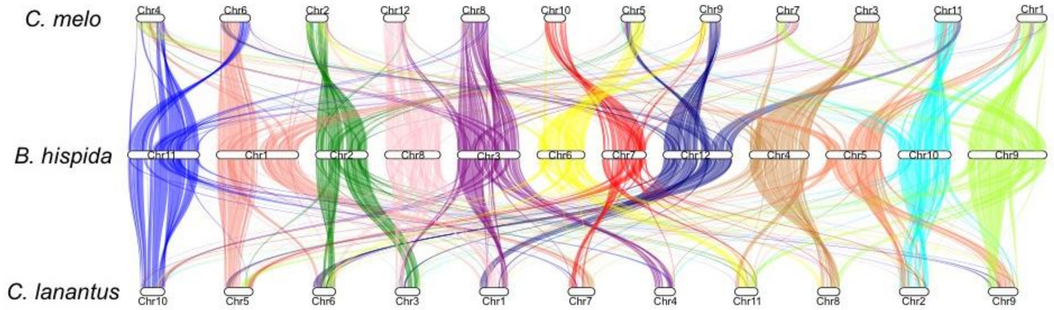

Supplementary Figure 9. Syntenic relationships across three genomes.

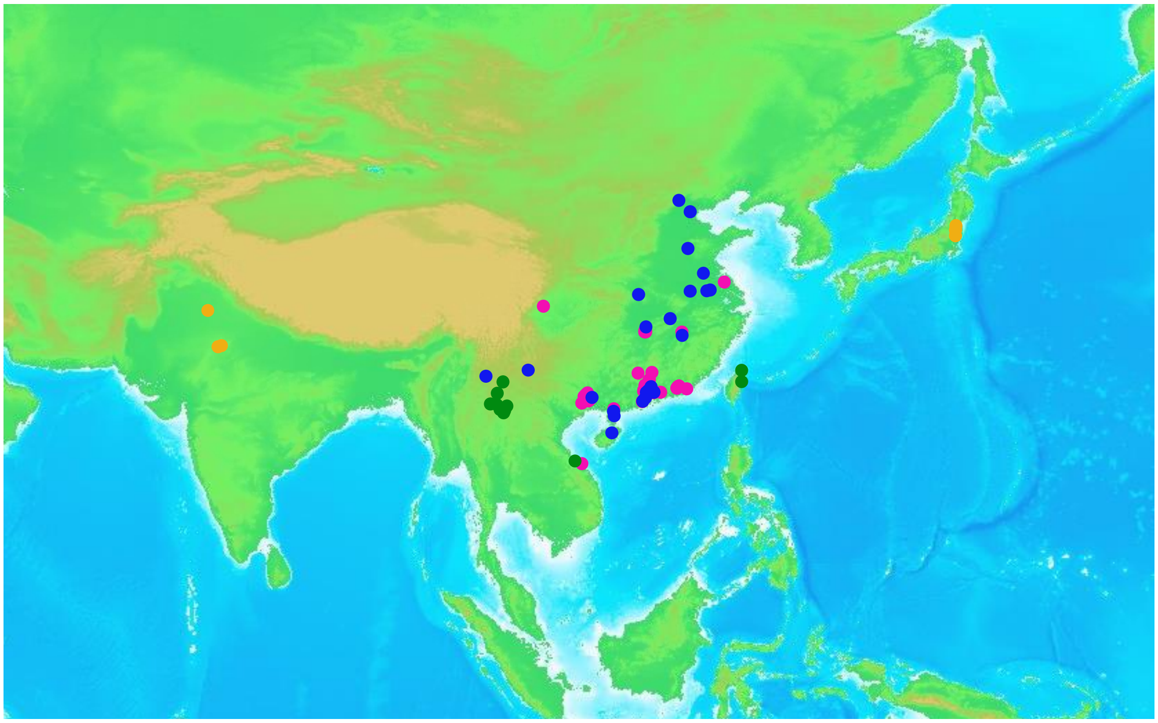

Supplementary Figure 10. The geographic distribution of the 146 accessions. Each accession is represented by a dot on the map. Magenta: cultivated wax gourds without wax; blue: cultivated ones with wax; green: Xishuangbanna landraces; orange: wild ones. The map was generated using the QGIS software (QGIS3.4; https://www.qgis.org/en/site/about/index.html).

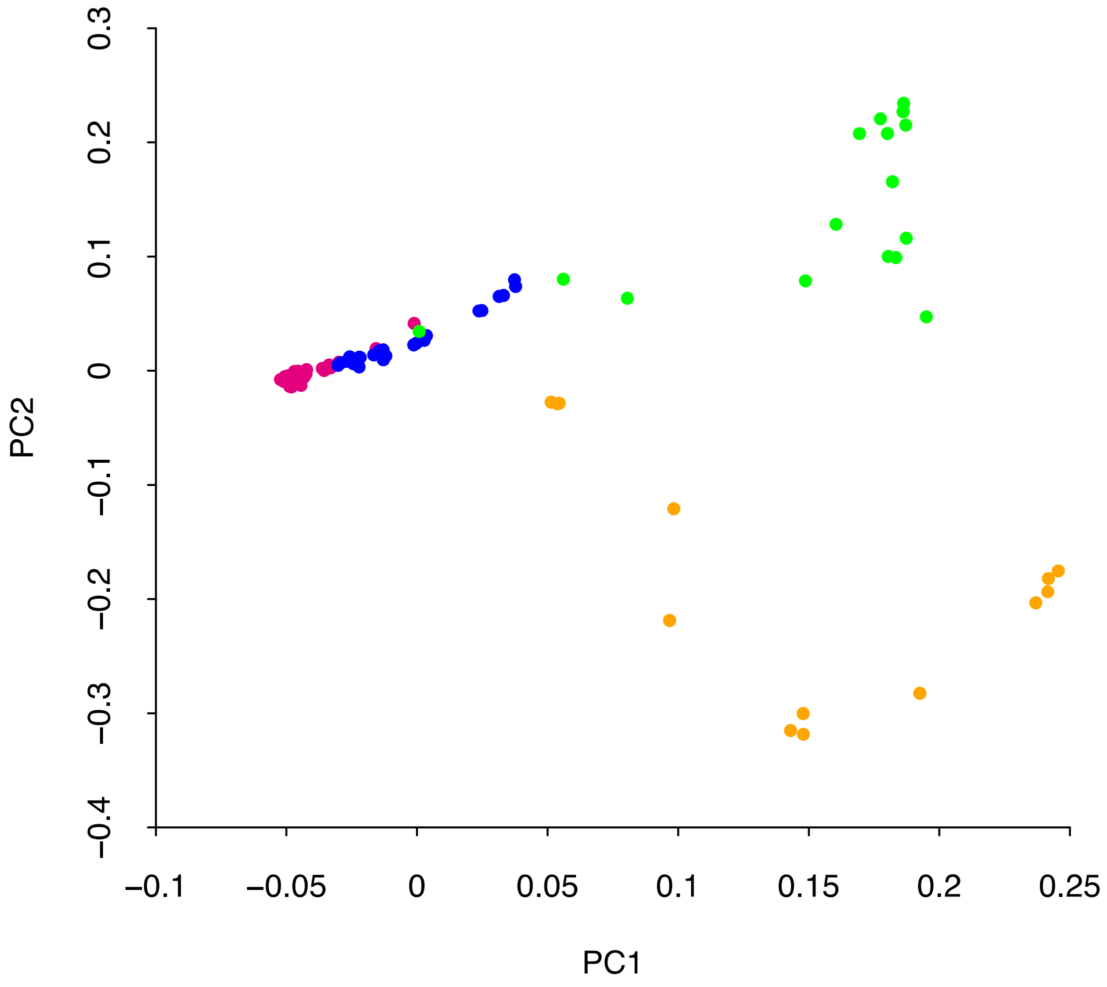

Supplementary Figure 11. Principal component analysis of the 146 wax gourd accessions. Source data are provided as a Source Data file.

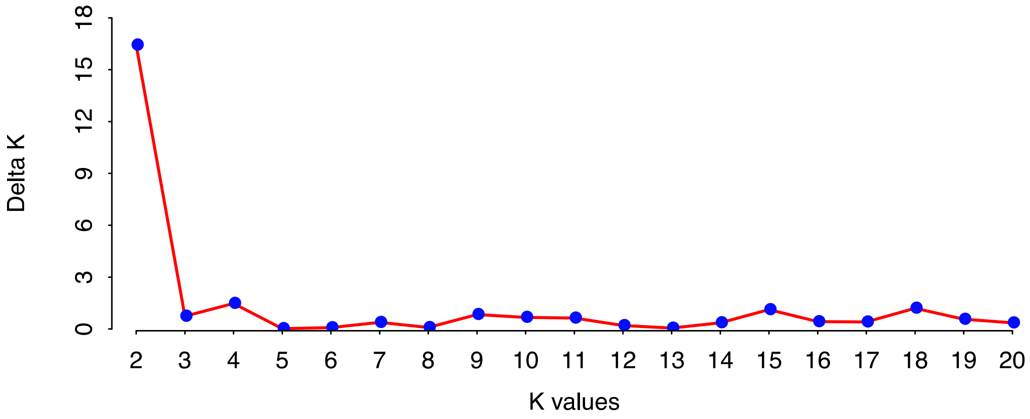

Supplementary Figure 12. Delta K analysis for the number of groups for the 146 wax gourd accessions.

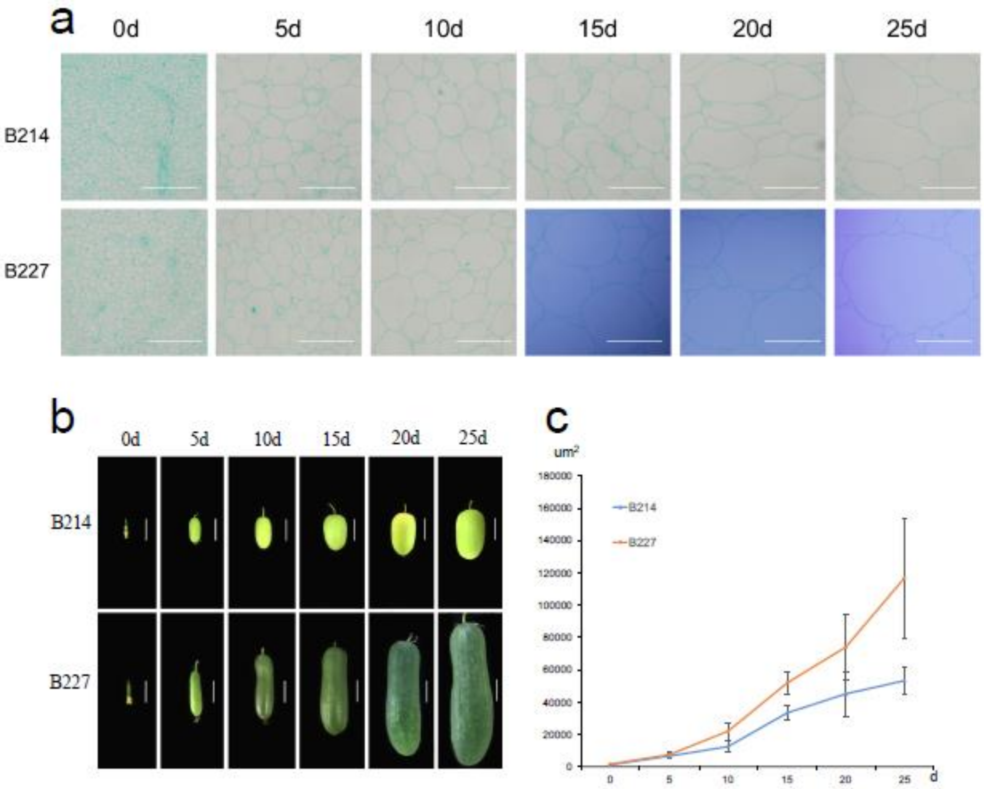

Supplementary Figure 13. Histological and morphology of wax gourd fruit. a. Morphology of cell size during fruit development Bar = 200um. **b.** Images of typical fruits during development, Bar = 10cm. **c.** Increase of cell volume from accession B214 and B227 from the longitudinal section. Error bars represent ± sd.

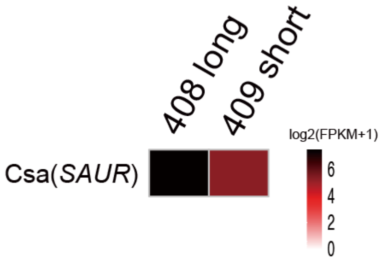

Supplementary Figure 14. Differential expression of SAUR gene in two cucumber near isogenic lines.

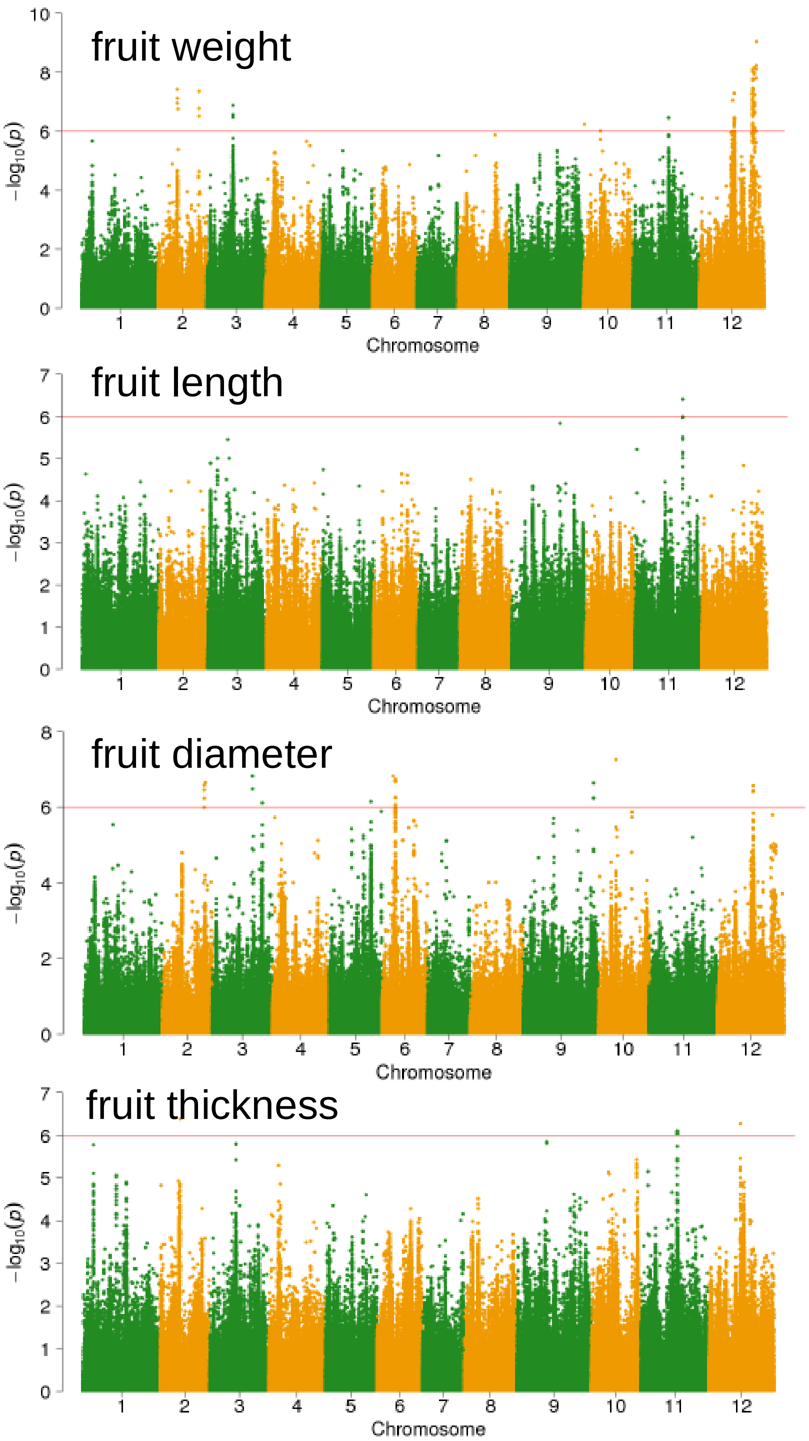

Supplementary Figure 15. GWAS results of fruit traits in wax gourd genome.

Supplementary Table 1. Summary of the sequencing data for wax gourd.

Library type	Library insert size	Data (Gb)	Read length		GC(%)		Q30	
			Read1	Read2	Read1	Read2	Read1	Read2
**Pair-end**	180bp	18.7	100	100	33.3	33.3	97	97
	500bp	1.0	100	100	36.3	36.3	98	92
	500bp	9.6	100	100	36.1	36.2	97	93
**Mate-pair**	2kb	0.9	91.6	91.4	38.5	38.6	95	93
	2kb	0.4	100	100	38.4	38.5	96	94
	2kb	9.4	100	100	38.6	38.7	95	96
	8kb	0.4	100	100	37.3	37.4	97	96
	8kb	1.0	93.2	92.9	37.0	36.8	88	85
**PacBio**		15						
	**total**	**55.4**						

Supplementary Table 2. Summary of gapped regions.

Chromosome	Size of N base (bp)
**chr10**	947,933
**chr11**	1,254,718
**chr12**	1,099,274
**chr1**	1,359,133
**chr2**	1,029,499
**chr3**	1,068,153
**chr4**	1,109,909
**chr5**	955,781
**chr6**	914,502
**chr7**	907,125
**chr8**	1,033,663
**chr9**	1,498,830
**contigs**	1,600,148
**Total**	14,778,668

Supplementary Table 3. Summary of contigs.

Contig	Size(bp)
**Total length**	898,172,958
**Count**	26,315
**Average**	34,132
**Median**	19,501
**N00**	479,328
**N10**	164,196
**N20**	126,004
**N30**	101,266
**N40**	83,251
**N50**	68,515
**N60**	55,048
**N70**	42,867
**N80**	30,679
**N90**	18,560
**N100**	349

Supplementary Table 4. Summary of predicted repeat sequences in the wax gourd genome.

Repeat sequence type		Copy number	Sequence length (Mb)	Percentage (%)
**Dispersed repeat**	**DNA transposable elements**	**310,469**	**94.06**	**10.30**
	CMC	18,688	8.56	0.94
	hAT	42,093	13.78	1.51
	Helitron	1,132	0.32	0.04
	MuLE	46,675	19.19	2.10
	PIF	10,903	3.25	0.36
	TcMar	87	0.008	0.001
	other	190,891	48.96	5.36
	**RNA transposable elements**	**769,754**	**545.39**	**59.73**
	non-LTR RT			
	LINE	57,565	16.20	1.77
	SINE	11,350	1.10	0.12
	LTR RT			
	Copia	376,614	329.41	36.08
	Gypsy	201,768	142.45	15.50
	other	122,457	56.22	6.12
	**Unknown transposable elements**	**44,510**	**12.95**	**1.42**
**Tandem repeat**	**Microsatellite**	**34,509**	**1.48**	**0.16**
	**Minisatellite**	**301,262**	**23.91**	**2.62**
	**Satellite**	**21,343**	**11.72**	**1.28**

Supplementary Table 5. Functional annotation of the wax gourd protein-coding genes.

Type	Number	Percentage (%)
**Total**	27,467	100
**NR**	21,002	76.46
**InterPro**	17,767	64.68
**Swiss-port**	20,770	75.62
**Arabidopsis**	20,480	74.56
**GO**	17,767	64.68
**Annotated**	21,227	77.28

Supplementary Table 6. NBS-LRR genes in different genomes.

Species	Number	Full NB - ARC domain
**Cucumber**	74	43
**Melon**	84	56
**Wax gourd**	82	59
**Water melon**	49	34
**Bitter gourd**	78	48
**Soybean**	164	NA
**Arabidopsis**	251	NA
**Grape**	330	NA
**Tomato**	265	NA
**Rice**	535	NA

NA: Not analyzed

Supplementary Table 7. Comparisons of gene models between the wax gourd and the other three *Cucurbitaceae* species.

	Wax gourd	Cucumber	Melon	Watermelon
**Genome size(Mb)**	913	196	407	355
**Gene number**	27,467	24,274	27,427	23,440
**Mean gene size(bp)**	3,962	3,674	2,776	2,770
**Mean CDs size(bp)**	1,098	1,149	950	1,109
**Mean exon size(bp)**	328	276	271	242
**Mean exon number/gene**	6.6	7.1	4.6	4.6
**Mean intron size(bp)**	1,044	814	506	654

Supplementary Table 8. Comparisons of the repeats content.

RT_type	Watermelon	Cucumber	Melon	Wax gourd
**DNA_RT**	6.35	22.07	27.46	94.06
**LINE**	10.43	10.26	4.52	16.20
**SINE**	0.01	0.49	0.29	1.10
**LTR_Copia**	35.06	15.99	38.39	329.41
**LTR_Gypsy**	20.48	12.53	25.16	142.45
**LTR_other**	28.97	8.74	3.92	56.22
**Unknown**	32.74	1.02	26.60	12.95

Note: repeat content: Mb.
