# Supplementary material for: The wax gourd genomes offer insights into the genetic diversity and ancestral cucurbit karyotype
Source: Nat Commun. 2019 Nov 14;10:5158. doi: 10.1038/s41467-019-13185-3 (PMC6856369; doi:10.1038/s41467-019-13185-3)
Supplement: Supplementary file 3 — Reporting Summary [file 41467_2019_13185_MOESM3_ESM.pdf]

nature research

Corresponding author(s): Zhonghua Zhang, Biao Jiang

Last updated by author(s): Oct 21, 2019

Reporting Summary

Nature Research wishes to improve the reproducibility of the work that we publish. This form provides structure for consistency and transparency in reporting. For further information on Nature Research policies, see Authors & Referees and the Editorial Policy Checklist .

Statistics

For all statistical analyses, confirm that the following items are present in the figure legend, table legend, main text, or Methods section.

n/a	Confirmed	
☐	☒	The exact sample size (*n*) for each experimental group/condition, given as a discrete number and unit of measurement
☐	☒	A statement on whether measurements were taken from distinct samples or whether the same sample was measured repeatedly
☐	☒	The statistical test(s) used AND whether they are one- or two-sided<br>*Only common tests should be described solely by name; describe more complex techniques in the Methods section.*
☒	☐	A description of all covariates tested
☐	☒	A description of any assumptions or corrections, such as tests of normality and adjustment for multiple comparisons
☐	☒	A full description of the statistical parameters including central tendency (e.g. means) or other basic estimates (e.g. regression coefficient) AND variation (e.g. standard deviation) or associated estimates of uncertainty (e.g. confidence intervals)
☐	☒	For null hypothesis testing, the test statistic (e.g. *F*, *t*, *r*) with confidence intervals, effect sizes, degrees of freedom and *P* value noted<br>*Give P values as exact values whenever suitable.*
☐	☒	For Bayesian analysis, information on the choice of priors and Markov chain Monte Carlo settings
☒	☐	For hierarchical and complex designs, identification of the appropriate level for tests and full reporting of outcomes
☐	☒	Estimates of effect sizes (e.g. Cohen's *d*, Pearson's *r*), indicating how they were calculated

Our web collection on statistics for biologists contains articles on many of the points above.

Software and code

Policy information about availability of computer code

Data collection	TIGR plant repeats database (http://plantrepeats.plantbiology.msu.edu),were used to mask the repeats in the wax gourd genome. non-redundant plant protein sequences downloaded from Uniprot (http://www.uniprot.org) for homologous gene annotation. No software for data collection was used.
Data analysis	All of softwares used in this study have been fully described including specifying versions in the Methods.

For manuscripts utilizing custom algorithms or software that are central to the research but not yet described in published literature, software must be made available to editors/reviewers. We strongly encourage code deposition in a community repository (e.g. GitHub). See the Nature Research guidelines for submitting code & software for further information.

Data

Policy information about availability of data

All manuscripts must include a data availability statement. This statement should provide the following information, where applicable:

- Accession codes, unique identifiers, or web links for publicly available datasets
- A list of figures that have associated raw data
- A description of any restrictions on data availability

The wax gourd genome sequences have been deposited in Genome Warehouse, in the BIG Data Center, with accession number GWH000054; and in GenBank, in NCBI, with BioProject ID PRJNA430006. The raw sequenced reads from the 146 wax gourd accessions have been deposited in the Genome Sequence Archive, in the BIG Data Center, under accession number CRA001259; and in the sequence read archive (SRA), in NCBI, under accession number SRP224893. The raw transcriptome sequences have been deposited in the Genome Sequence Archive, in the BIG Data Center, under accession number CRA001814; and in the SRA, in NCBI, under accession number SRP224600. The source data underlying Figures 1a, 3a-d, 4b, c and 5a, b, d, as well as Supplementary Figures 1, 4, 7, 8 and 11 are provided as a Source Data file.

Field-specific reporting

Please select the one below that is the best fit for your research. If you are not sure, read the appropriate sections before making your selection.

- [x] Life sciences
- [ ] Behavioural & social sciences
- [ ] Ecological, evolutionary & environmental sciences

For a reference copy of the document with all sections, see nature.com/documents/nr-reporting-summary-flat.pdf

Life sciences study design

All studies must disclose on these points even when the disclosure is negative.

Sample size	An inbred wax gourd line, B227, was selected for genome sequencing. 146 wax gourd accessions including the reference genome B277 were selected for variation analysis.
Data exclusions	No sample was excluded in all analyses procedure. SNP set pre-filtering of population analysis was described in the Methods section.
Replication	Transcriptome sequencing analyses was conducted on three replicates for each tissue.
Randomization	NA
Blinding	NA

Reporting for specific materials, systems and methods

We require information from authors about some types of materials, experimental systems and methods used in many studies. Here, indicate whether each material, system or method listed is relevant to your study. If you are not sure if a list item applies to your research, read the appropriate section before selecting a response.

Materials & experimental systems

n/a	Involved in the study	
[ ]	[x]	Antibodies
[x]	[ ]	Eukaryotic cell lines
[x]	[ ]	Palaeontology
[x]	[ ]	Animals and other organisms
[x]	[ ]	Human research participants
[x]	[ ]	Clinical data

Methods

n/a	Involved in the study	
[x]	[ ]	ChIP-seq
[x]	[ ]	Flow cytometry
[x]	[ ]	MRI-based neuroimaging

Antibodies

Antibodies used	Please see the Immunoblotting part in method section
Validation	This anti-MYC monoclonal antibody is produced by immunizing animals with a synthetic peptide (KLH-coupled) corresponding to residues 410-419 of human c-Myc (EQKLISEEDL). Myc-Tag (19C2) Mouse mAb detects over-expressed or recombinant proteins containing the Myc epitope tag. An examples of a reporter using the antibody in plant for IP is Zupei Liu, Gang Chen, Fan Gao, Ran Xu, Na Li, Yueying Zhang, Yunhai Li. Transcriptional Repression of the APC/C Activator Genes CCS52A1/A2 by the Mediator Complex Subunit MED16 Controls Endoreduplication and Cell Growth in Arabidopsis. The Plant Cell. Published June 2019. DOI:https://doi.org/10.1105/tpc.18.00811
