# Supplementary material for: The wax gourd genomes offer insights into the genetic diversity and ancestral cucurbit karyotype
Source: Nat Commun. 2019 Nov 14;10:5158. doi: 10.1038/s41467-019-13185-3 (PMC6856369; doi:10.1038/s41467-019-13185-3)
Supplement: Supplementary file 4 — Description of Additional Supplementary Files [file 41467_2019_13185_MOESM4_ESM.docx]

**Description of Additional Supplementary Files**

File name: Supplementary Data 1
Description: Expanded gene families in wax gourd genome.

File name: Supplementary Data 2
Description: List of the resequenced wax gourd accessions.

File name: Supplementary Data 3
Description: Number of different types of SNPs.

File name: Supplementary Data 4
Description: Summary of domestication sweep regions.

File name: Supplementary Data 5
Description: The genes within domestication sweep regions.

File name: Supplementary Data 6
Description: Summary of improvement sweep regions.

File name: Supplementary Data 7
Description: The genes within improvement sweep regions.

File name: Supplementary Data 8
Description: Physical intervals of fruit-related QTLs.

File name: Supplementary Data 9
Description: Physical intervals of GWAS signals for fruit-related traits.

File name: Supplementary Data 10
Description: RNA-seq data of B227(big fruit) and B214(small fruit) in three different developmental stages.

File name: Supplementary Data 11
Description: Phenotypic data for fruit-related traits of 146 accessions.
