# Supplementary material for: The wax gourd genomes offer insights into the genetic diversity and ancestral cucurbit karyotype
Source: Nat Commun. 2019 Nov 14;10:5158. doi: 10.1038/s41467-019-13185-3 (PMC6856369; doi:10.1038/s41467-019-13185-3)
Supplement: Supplementary file 2 — Peer Review [file 41467_2019_13185_MOESM2_ESM.pdf]

First round of review

Reviewers' comments:

Reviewer #1 (Remarks to the Author):

The current manuscript describe a comprehensive genomic effort focused on wax gourd and its relation with other cucurbit species. The authors started by creating a draft de novo assembly of the genome of this plant, which may be used as a first reference genome for this species. Then they performed various comparative and evolutionary analyses to describe ancestry and evolutionary processes across cucurbit species. Finally, they describe the population structure and genetic variation across wax gourd diverse collection and the mapping of fruit morphology traits. Assembly of a genome for additional cucurbits species is valuable for the evolutionary analysis of this important family and will provide valuable data for that purpose. Assembly of a reference genome for *Benincasa hispida* is of interest to this crop community and will encourage and facilitate further and more effective genetic research in this species.

While this manuscript includes comprehensive genomic information, its major weakness is in the part of the trait mapping data and the associations that are proposed between genetic variation and fruit morphology traits. In that respect, the claim in the abstract ("we found that genes involved in plant hormone signaling and cell cycle regulation likely contribute to the large fruit size ") is an overstatement as it is supported by weak experimental results in the manuscript.

Response:

Thanks for your comments. To facilitate the identification of the candidate genes for fruit size, we have now added the RNA-seq data of fruit at three (0,10 and 20 days after pollination[DAP]) developmental stages for both wax gourd accession B227 bearing large fruit and B214 with small fruit. The differentially expressed genes (DEGs) were identified and analyzed, and in total, 1,642, 4,320 and 4,307 genes were identified as DEGs (Response Data 1-3) at 0, 10 and 20 DAP, respectively (Response Fig.1). To avoid confusion and overstatement, we have also rephrased the abstract.

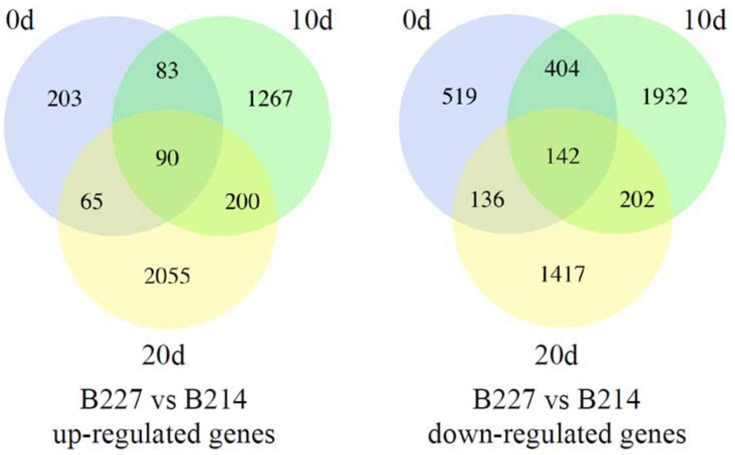

Response Fig. 1 Overlap of differentially expressed genes between large (B227) and small (B214) fruited-accession across three fruit developmental stages.

Specific comments:

1. Supplementary Fig. 3 show the relation between genetic and physical maps across the 12 wax gourd chromosomes. The pattern is almost linear across all chromosomes. This is very uncommon as there is usually variation in recombination frequency within chromosomes and in particular low recombination rate at the centromeric regions. This is not reflected here and require explanation. Also on this figure, what are the white and gray patterns on the chromosomes bellow each box?

Response:

Although the relation between genetic and physical maps is almost linear, we do observe several regions showing low recombination, such as the region on the chromosome 5. As mentioned in the manuscript, the wax gourd genome could have retained the most ancestral cucurbit karyotype. It has a particularly large genome, and the repetitive sequences were greatly expanded compared with other cucurbits. This implies that wax gourd genome may have a special genome organization. The genetic and physical map were significantly consistence, indicating the high degree of accuracy of the assembly.

The white and gray patterns on the chromosomes indicate different scaffolds. The information was added in the Supplementary Fig. 3.

2. The table to the right of Fig. 1a needs a legend explanation.

Response:

We added a legend for the table to the right of Fig. 1a.

3. Figure 2a is complicated and visually challenging. In addition, it needs to be described a bit more either through methods or at the results. It was unclear to me how ancestral chromosomes were determined.

Response:

To clarify the process to infer the ancestral chromosomes, more detailed information was included in the Methods section of the revised manuscript.

4. In Fig. 4c, not clear what is the purpose of plotting K=2,3,4 and not just k=4 which the best clustering model that is proposed.

Response:

Yes, k=4 is the best clustering model. However, if only the model of k=4 is shown, the relationship between the accessions of the Landrace group and that of the Wild group would not be clear. To help better understand the relationships of these accessions, we presented the results of different cluster numbers (K = 2, 3 and 4).

5. Reduction of nucleotide diversity to detect candidate regions of domestication and improvement - It is reported here that 10%-15% of the wax gourd genome show signature of selection. These high percentages that are distributed across the whole genome (Fig. 5) are reducing the ability to use this parameter as an effective method to detect candidate genes associated with domestication or improvement, in particular not as a stand-alone parameter. Lines 220-225 describe a candidate gene (Bhi10G001538) based on selection sweep in a 500 Kb region. The only other supportive information was the fact that this gene is highly expressed in fruit. This is very speculative and not clear why this example is provided as there is no

QTL reported in this interval, or differential expression analysis of this gene between large and small-fruited accessions.

Response:

Thanks for your comments. We have attempted to use several cutoffs (top 1%, 5%, 10%) to detect the sweep regions. Using top 1% or 5% as cutoff, several QTL regions (*fw3.1*, *fd3.1*and *ft3.1*) related to fruit size in wax gourd were excluded, but they show obvious sweep signal (see Fig. 5b). Therefore, we selected the top 10% as the cutoff, as was done for maize[1] and pear[2]. To improve the identification efficiency of domestication and improvement sweeps, we further calculated the XP-CLR scores, and retained those with top 50% of XP-CLR scores, as with apple[3] and pear[2] (Response Table 1).

To provide more evidence for the candidate gene *Bhi10G001538*, we added RNA sequencing (RNA-seq) analysis between large- and small-fruited accessions. As expected, *Bhi10G001538*, encoding a small auxin-up-regulated RNA (SAUR), is significantly up-regulated in large-fruited accession, at three developmental stages, especially at 10 DAP compared to that in small-fruited accession (Fig. 5c). These data provide support for the hypothesis that *Bhi10G001538* contributes to fruit size.

Response Table1. Summary of sweep regions in the wax gourd genome

	Domestication	Improvement
Number of sweep regions	234	168
Max sweep region	2,580 kb	2,440 kb
Min sweep region	200 kb	200 kb
Average sweep region	456 kb	475 kb
Total sweep region length	106.7 Mb	80.3 Mb
Ratio of assembly genome	11.60%	9.10%
Number of genes	3,939	2,251

6. Line 230: "cytological" should probably be replaced with histological.

Response:

Thanks for this suggestions. We used the word "histological" to replace "cytological"

in the revised manuscript.

7. Supplementary Fig. 13: I could not find information on the histological and the cell volume analyses. Should be added to the materials and methods.

Response:

We have the following to the Methods section of the revised manuscript:

To measure cell size in the wax gourd fruit of B227 and B214, 1-cm-thick sliced samples were cut from the outer, middle, and inner pericarp at different developmental time points, at 0, 5, 10, 15, 20 and 25 days after pollination (DAP). These sections were fixed in a solution of ethanol (70%), acetic acid, and formaldehyde (90:5:5 by volume) and then embedded into paraffin. Subsequently, 8 µm-thick-microtome sections were prepared (from cross- and longitudinal, stained with haematoxylin-eosi, and examined and images collected by light microscopy. Cell size in each section was calculated by ImageJ software; the top 30 cells in size were counted, and the mean and variance in cell size calculated, for each development period examined. Measurements were made at 3 different sites of each tissue, for 3 sections from each fruit.

8. Fig. 4b: accession names bellow the graph are too small to read. Lines 232-233 are referring to this figure with respect to accession B214 but I could not use this reference.

Response:

Accession names are listed in Supplementary table 2 according to the orders in Fig. 4b. Accession B214 is highlighted in Fig. 4b.

9. QTL mapping (Lines 226-244): this part is based on previously published data. The authors refer to a previous QTL mapping study (Liu W, et al. Genetic analysis and QTL mapping of fruit-related traits in wax gourd (Benincasa hispida). Euphytica) but a short sentence referring to the population used is lacking and essential (size, generation, etc…). Also a QTL table from this linkage population is not shown anywhere and should be added (as supplementary). In this part, QTL

fw3.1 is described and shown to be located within a domestication sweep interval, and a candidate gene (Bhi03G000723) is proposed based on annotated function. The authors also refer to the high expression in fruit of this gene but this is not reflected in Fig. 5f as the expression looks uniform also in leaf and root. In line 243, "Fig. 6f" should be corrected to Fig. 5f. It is not mentioned whether this QTL also showed on the GWAS analysis. Taken together this is a speculative discussion on a candidate gene

– the co-occurrence of QTL within domestication sweep interval is very probable to be random considering the high proportion of domestication sweep regions.

Response:

We used an F2 segregating population including 146 individuals which were derived from a cross between landrace accession B214, with fruit of ~2.0 Kg, and cultivated accession ‘B227’ with fruit of ~20 Kg. This information has been incorporated into our revised manuscript. The physical intervals of these QTLs have been presented in Supplementary data 8. For the candidate gene, *Bhi03G000723*, we identified five missense variant SNPs in its coding region. However, as mentioned by Reviewer 1, the level of analysis of this candidate gene is limited, and thus any conclusion as to function remains speculative. In view of this fact, we chose a new example candidate gene having more supportive evidence.

10. The authors can use syntheny analyses to identify and focus on candidate genes from other cucurbits. Specifically, there are several QTL studies on fruit size and shape QTLs in melon, including the mapping of candidate genes from other species (i.e. tomato). Alignment of QTLs in syntenic regions could add another layer to candidate genes identification.

Response:

In response to your suggestions, we collected the genes responsible for fruit size in tomato and other cucurbits. By aligning protein sequences of these genes against the genes within sweep and fruit size-related QTL regions in wax gourd, an orthologous

(*Bhi10G000196*) gene was identified. *Bhi10G000196* is located at the physical interval of the *fl10.1* QTL for fruit length and the domestication sweep from 5.2 Mb to 6.5 Mb. The mutation of its homologue *SlFIN* (*Solyc11g064850*) in tomato can cause enlarged fruit[4]. RNA-seq data showed that *Bhi10G000196* is down-regulated in the large-fruited accession, at three developmental stages (Response Fig. 2a), compared to that in small fruited-accession, supporting a role in enlarged fruit during wax gourd domestication. This gene, as a new candidate gene with more evidence, was added into the revised manuscript.

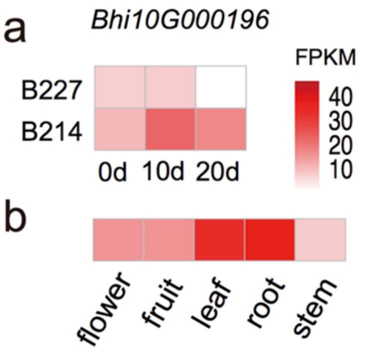

Response Fig. 2 Expression profiles of a new candidate gene (*Bhi10G000196*) involved in wax gourd fruit size.

11. GWAS is described in lines 245-253. Again, in this part, the co-localization of GWAS hits with domestication/improvement sweeps has high probability and could occur by chance alone. There is no description or definition of co-localization parameters (how is the confidence interval for GWAS hit defined?). The candidate gene mentioned in this part (Bhi11G001327) is also indicated within a QTL interval (based on Fig 5b but not in the text). Is that the case? it's not mentioned what is the QTL interval size and whether this gene indeed located within it?

Response:

The confidence intervals for our GWAS results are defined on the basis of p-values,

and this has been added in the revised manuscript.

The candidate gene *Bhi11G001327* is indeed within the interval of QTL fd11.1. To avoid undue speculation, in the revised manuscript, we provide only a list of genes with significant signals.

12. Fig. 5f show expression profile of three genes that are described as candidates for association with fruit size. The legend must be expanded for this figure: what is the scale, what is the reference expression used for the relative analysis. I could not find any description of this analysis anywhere (not in the legend or in the methods section. Which genotype was used? What expression analysis technique was used (RNA-Seq, qRT-PCR)? Number of reps? Statistics? etc…

Response:

We revised this figure and added detail information into the legend. Expression profiles for the candidate genes were generated by RNA-seq.

13. Analysis of differential expression of candidate genes between large and small fruited-accession is required to further support the claimed hypothesis on candidate genes.

Response:

As suggested, we have added the RNA-seq data of fruit at three (0, 10 and 20 DAP) developmental stages for large (B227) and small (B214) fruited accessions. DEGs between them were identified, using a false discovery rate (FDR) < 0.05 and fold change (FC) > 2 as cutoffs. In total, 1,642, 4,320 and 4,307 genes were identified as DEGs (Response Data 1-3) at 0, 10 and 20DAP, respectively (Response Fig. 1). *Bhi10G001538*, encoding a small auxin-up-regulated RNA (SAUR), and a new identified gene, *Bhi10G000196*, are supported by our differential expression analysis.

14. The discussion is very thin and should either be integrated with results or expanded.

Response:

As suggested, we have integrated the Discussion with the Results section.

15. Line 259: TE is used the first time as acronyms. Full term should be used.

Response:

Full term for TE was used.

16. Line 269: the verb "prefer" should probably be replaced with a passive verb that describe evolutionary advantage.

Response:

We combined the Results and Discussion sections in the revised manuscript and this sentence was rephrased.

17. In the variant calling description at the methods section it is not mentioned what minor allele frequency (MAF) was used as filtering criteria.

Response:

Minor allele frequency (MAF) was more than 0.05 and has been indicated in the revised manuscript.

18. In the GWAS description in the methods section (lines 407-413), three phenotyping seasons are mentioned but it is not clear how the data was used for association analysis. What was the correlation between seasons? Was the GWAS performed for each season separately? Were the same QTLs identified across seasons? or, was the data integrated and used for mapping?

Response:

Correlations of fruit-related traits between different years are from 0.990 to 0.998 (Response Data 4), indicating the high consistency across years. Therefore, the average values of fruit weight, length, diameter and fruit thickness were used in our GWAS. We also performed GWAS based on data of each season (2014, 2015, 2016), and similar results were obtained. The Methods section has been rewritten to clarify how these phenotypic data were assessed.

19. The resequencing of diverse accession resulted in identification thousands of missense, nonsense and splice-sites mutations across the genome (lines 187-188). Was this information used to asses candidate genes in QTL intervals?

Response:

Yes, this information was used to assess candidate genes in sweeps and QTL intervals. For example, within the physical interval of the *fl10.1* QTL for fruit length, one domestication sweep from 5.20 to 6.56 Mb contained 55 genes. Among these genes, *Bhi10G000196* is a homologue of *SlFIN* (*Solyc11g064850*) in tomato, the mutation of which can cause enlarged tomato fruit[4]. Moreover, differential expression analysis supports its role in affecting fruit size. To provide further evidence, we examined the SNPs within the genic regions. A total of three missense variants and six synonymous variants were identified. These variants are only present in wild accessions, implying their selection during domestication. (Response data 5)

Reviewer #2 (Remarks to the Author):

The manuscript describes a draft genome assembly of wax gourd and genome variations derived from resequencing dat of 146 wild, landrace and cultivated wax gourd accessions. Through comprehensive comparative and population genomic analyses, the authors concluded that the wax gourd genome represents the most ancestral karyotype of cucurbits, and they identified potential genome regions that have been affected during wax gourd domestication and improvement, as well as candidate genes contributing to large fruit size of wax gourd. The reported wax gourd draft genome provides a valuable resource for future comparative and evolutionary genomic studies, and the study provides certain insights into cucurbit genome evolution and wax gourd domestication.

Major:

1. The title is quite misleading and inaccurate. First, the genome does not bear a giant fruit. Second, the sequenced wax gourd genome is not an ancestral cucurbit

genome; it could have just retained the most ancestral cucurbit karyotype. This needs to be fixed throughout the entire manuscript.

Response:

Thanks for your insightful comments. The title has been revised to “The wax gourd genomes offer insights into the ancestral cucurbit karyotype and the genetic basis of diversity”. The revised manuscript has been written to reflect this situation.

2. Genome assembly: The authors need to provide the statistics of assembled contigs as well as the size of gapped regions.

Response:

We added this information in the revised manuscript (Supplementary Table 2 and 3).

3. Cucurbit ancestral genome reconstruction (Page 6-7 and Page 15): First, the method on ancestral genome reconstruction is poorly written (Line 369-373) and lacks sufficient details.

Response:

We added more detailed information on ancestral genome reconstruction in the Methods section on “Evolutionary scenario of cucurbit genomes”, as follows:

By comparing different cucurbit genomes, phylogenetically, we adopted a bottom-up approach to reconstruct the ancestral cell karyotypes of cucurbit plants. Firstly, by inferring putative homologous genes and collinear genes, we drew homologous gene dot plots within a genome and between genomes. Ks values were estimated to infer collinear genes produced by different events, and the information was integrated into the dot plots. Secondly, in that pumpkin is the outgroup of other studied cucurbits, we checked the dot plots to assess whether its chromosomes or main structures of its chromosomes, were shared by other cucurbit plants. Thirdly, the fusion and fission events during genome evolution of cucurbit species from their ancestral chromosomes were determined.

Then in the Results and Discussion we revised the section on ancestral chromosomes reconstruction. Actually, we found that, ignoring some intra-chromosome breakages

and inversions, seven wax gourd chromosomes, ., were shared with at least one of the other cucurbits, showing that they are most likely proto-chromosomes before the divergence of these cucurbits, ordinally named proto-chromosomes 1-7. For an extra whole-genome duplication in pumpkin, if one copy of a duplicated chromosome is shared with other cucurbits, this would mean that it represents a proto-chromosome in the cucurbit common ancestor. We determined that wax gourd and pumpkin share six out of these 7 proto-chromosomes, suggesting the conserved nature of their genomes. Furthermore, for the wax gourd chromosomes, Bhi2, Bhi3, and Bhi10, both homoeologous copies produced by the cucurbit-common whole-genome duplication was preserved in pumpkin (Cma), showing that they could represent proto-chromosomes before the event.

Large patches of chromosome segments shared by extant genomes can be used to infer

other proto-chromosomes. Some of these large patches lack linked co-existence with other chromosomes or chromosome regions. For example, wax gourd chromosome Bhi 9 could be found to occur in partite manner in other genomes, and each part is independent of the other one, and at the mean time independent of other chromosomes; this shows their independence in the ancestral genome, and leads to the definition of proto-chromosomes 8 and 9. Similarly, we inferred proto-chromosomes 11, 12, 14, and 15. Some large patches have linked co-existence in extant genomes, showing that they could have originated from the same proto-chromosome. For example, one patch in Bhi 1 and another in Bhi 11 co-occurs in four genomes, especially two times in Cma, showing that they should be from the same proto-chromosome, even one being prior to the cucurbit-common whole-genome duplication; this leads to the inference of proto-chromosome 13. Similarly, we inferred proto-chromosome 10.

Second, the ancestral genome of cucurbits the authors reconstructed has 15 protochromosomes, which is different from the number of protochromosomes (12)

described in Wu et al. (https://onlinelibrary.wiley.com/doi/full/10.1111/tpj.13722). The authors need to provide an explanation of this inconsistency.

Response:

Wu et al. took 12 melon chromosomes as cucurbit-common proto-chromosomes. Actually, we can easily infer that several melon chromosomes cannot represent ancestral chromosomes. For example, melon chromosome 1 (Cme1) corresponds two or more chromosomes in the other four genomes [Bhi (Bhi5, Bhi9)、Lsi (Lsi2, Lsi3), Cla (Cla2, Cla9)、Cma (Cma17, Cma19; and Cma8, Cma11)] (Response Fig. 3). The finding that Cme1 preserves its main structure in cucumber chromosome 7 (Csa7) can be easily explained by a merge in their latest common ancestor. The other Cme chromosomes 5, 7, and 11 can also be inferred to be formed in its own lineage, or its close lineages.

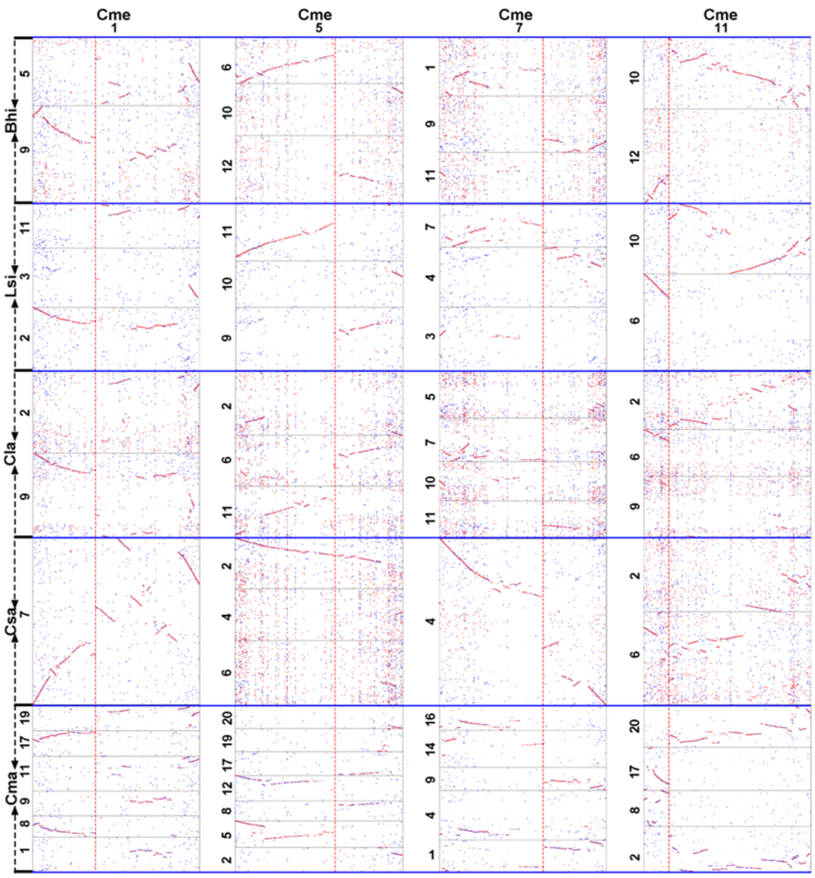

Response Fig. 3 Dotplot of the melon genome (Cme) compared to other cucurbits genomes.

Third, as indicated in their title of the manuscript, the authors were trying to emphasizing the significance of the wax gourd genome as an ancestral cucurbit genome. As I mentioned above, this is not correct. In addition, the significance that the wax gourd genome has retained the most ancestral cucurbit karyotype seems marginal.

Response:

We have rephrased the related content. The wax gourd genome has preserved the main structure of 7 proto-chromosomes, and other large patches, thus contributing to our understanding of the ancestral karyotype, demonstrating its conservativeness over other sequenced cucurbit genomes.

4. The “Discussion” section is very poorly written. The second paragraph of the Discussion is poorly developed. The hypothesis that cucurbit species with 12 chromosomes tend to retain the ancestral karyotype seems too speculative. In addition, there is no discussion at all related to the second part of the manuscript (genome variation, domestication and fruit size).

Response:

We have integrated the Results and Discussion sections in the revised manuscript. In addition, we have included more information related to genome variation and domestication.

5. Line 430-439: the authors identified domestication and improvement sweeps by scanning genomic regions with top 10% nucleotide diversity ratio. The threshold of top 10% seems to be very high - other similar studies generally use much more stringent thresholds. They should also use model-based approaches, such as

XP-CLR or similar methods. With the current method and parameter, the identified sweeps would contain quite a lot of false positives.

Response:

Thanks for your comments. This question was similar to the fifth question raised by Reviewer #1. We employed several cutoffs (top 1%, 5%, 10%) to detect the sweep regions. Using the top 1% or 5% as cutoff, several QTL regions (*fw3.1, fd3.1*and *ft3.1*) related to fruit size in the wax gourd were excluded, but they show obvious sweep signal (see Fig. 5b). Based on this finding, we selected the top 10% as the cutoff, as with maize[1] and pear[2]. To improve the identification efficiency of domestication and improvement sweeps, we further calculated the XP-CLR scores, and retained those with top 50% of XP-CLR scores, as in apple[3] and pear[2] (Response Table 1).

Minor:

6. Line 264-265, Population structure analysis shows that B214 is an admixture of wild, landrace and cultivated wax gourds (Fig. 4b): I could not see this from Fig. 4b.

Response:

B214 is highlighted in the revised manuscript.

7. Line 280, GWAS threshold of –log10(P)=6: how this cutoff was derived? How many SNPs were used for GWAS?

Response:

A total of 2,237,614 SNPs (MAF > 5% and Missing rate < 10%) for 146 accessions was used to perform the GWAS, and a threshold of –log10(P)=6 was set using N (the effective number of independent SNPs, P=1/N). The effective number of independent SNPs was calculated using Genetic type 1 Error Calculator (GEC) software[5]. This information has been included in the revised manuscript.

8. Line 352-353: which known divergence time(s) (between which species) in the tree was used to infer the divergence times?

Response:

Divergence times were estimated by the program MCMCtree in PAML (version 3.15) (http://abacus.gene.ucl.ac.uk/software/paml.html), based on known divergence time between cucumber and melon (about 10 MYA)[6]. This information was added to the revised manuscript.

9. Fig. 1 and Fig. 2 legends: please add the meanings of sWGD, CCT, ECH…

Response:

The abbreviations sWGD, CCT and ECH are specific whole genome duplication, cucurbit-common tetraploidization, and eudicot-common hexaploidization, respectively. This information was added in the revised manuscript.

10. Non-wax group and wax group could be compared to identify some interesting genome regions that may underlie the phenotypic difference.

Response:

Population fixation index (FST) between Cultivar2 (non-wax) and Cultivar1 (wax) group was calculated using HIERFSTAT[7] R package, base on the high confidence filtered SNPs (1,855,619), including 10,322 nonsynonymous SNPs. Highly differentiated regions of two Cultivar groups were identified, using 200 kb sliding windows with a step size of 20 kb with the top 5% of FST values. We detected 183 highly differentiated regions ranging from 200 kb to 2,280 kb in length (465 kb on average) (Response Data 6) including 2750 genes. In cucumber, two major QTL, *WP5.1* and *WP6.2* related to wax, were detected[8] (Response Data 7), but no QTL was defined in the wax gourd. We obtained a homolog set, including 301 genes, which are located in QTL *WP5.1* and *WP6.2*, based on syntheny analyses between cucumber and wax gourd. Only 11 genes overlapped between the homolog set and genes in differentiated regions (Response Data 8). In the absence of additional supportive evidence, we do not present these results in the revised manuscript.

Reviewer #3 (Remarks to the Author):

The article submitted to Nature Communications (manuscript number NCOMMS-19-00378) entitled ‘The sequence and variation of an ancestral cucurbit genome bearing giant fruit’, by Dasen Xie and collaborators delivers the genome sequence of wax gourd and investigates the evolution of the cucurbit genomes, the dynamics of repeated elements, the genomic footprint of domestication and improvement as well as delivers candidate genes driving fruit traits. While the article delivers a high quality reference sequence of the wax gourd genome, several major concerns and associated opened questions are addressed below:

Major concerns

Section ‘Identification of the cucurbit ancestral genome’:

-What calibration strategy/system has been used to infer speciation datation events in MYA from the 463 single-copy gene family?

Response:

This question is similar to the eighth question raised by Reviewer #2

Divergence times were estimated by the program MCMCtree in PAML (version 3.15) (http://abacus.gene.ucl.ac.uk/software/paml.html), based on the known divergence time between cucumber and melon (about 10 MYA)[6].

-Methods and tools for genome alignment, synteny inference and ancestral genome reconstruction are very sensitive to parameters and thresholds. The author should detail the (maybe arbitrary) parameters used in each steps, if any and possible impacts.

-What is mentioned in the method section as ‘using the most ancestral genome as reference the ancestral chromosome were inferred’ may seem too vague for readers to be able to reproduce the analysis.

- What can be the impact of using wax gourd as reference genome in defining ancestral karyotype that at the end appears similar to wax gourd? At least this point has to be mentioned that wax gourd was considered as reference genome. Methods

and tools and associated parameters are now available to reconstruct ancestral genomes and infer evolutionary scenario without any assumption of considering extant genomes as reference.

-In the text and associated supplementary figures mainly pumpkin/wax gourd/melon and pumpkin/wax gourd/melon comparisons have been considered. How the other pairwise comparisons have been integrated and exploited in the synteny identification?

Response:

We added more detailed information on ancestral genome reconstruction in the Methods section on “Evolutionary scenario of cucurbit genomes”, as follows:

By comparing different cucurbit genomes, phylogenetically, we adopted a bottom-up approach to reconstruct the ancestral cell karyotypes of cucurbit plants. Firstly, by inferring putative homologous genes and collinear genes, we drew homologous gene dot plots within a genome and between genomes. Ks values were estimated to infer collinear genes produced by different events, and the information was integrated into the dot plots. Secondly, in that pumpkin is the outgroup of other studied cucurbits, we checked the dot plots to assess whether its chromosomes or main structures of its chromosomes, were shared by other cucurbit plants. Thirdly, the fusion and fission events during genome evolution of cucurbit species from their ancestral chromosomes were determined.

Then in the Results and Discussion we revised the section on ancestral chromosomes reconstruction. Actually, we found that, ignoring some intra-chromosome breakages and inversions, seven wax gourd chromosomes, ., were shared with at least one of the other cucurbits, showing that they are most likely proto-chromosomes before the divergence of these cucurbits, ordinally named proto-chromosomes 1-7. For an extra whole-genome duplication in pumpkin, if one copy of a duplicated chromosome is shared with other cucurbits, this would mean that it represents a proto-chromosome in the cucurbit common ancestor. We determined that wax gourd and pumpkin share six

out of these 7 proto-chromosomes, suggesting the conserved nature of their genomes. Furthermore, for the wax gourd chromosomes, Bhi2, Bhi3, and Bhi10, both homoeologous copies produced by the cucurbit-common whole-genome duplication was preserved in pumpkin (Cma), showing that they could represent proto-chromosomes before the event.

Large patches of chromosome segments shared by extant genomes can be used to infer

other proto-chromosomes. Some of these large patches lack linked co-existence with other chromosomes or chromosome regions. For example, wax gourd chromosome Bhi 9 could be found to occur in partite manner in other genomes, and each part is independent of the other one, and at the mean time independent of other chromosomes; this shows their independence in the ancestral genome, and leads to the definition of proto-chromosomes 8 and 9. Similarly, we inferred proto-chromosomes 11, 12, 14, and 15. Some large patches have linked co-existence in extant genomes, showing that they could have originated from the same proto-chromosome. For example, one patch in Bhi 1 and another in Bhi 11 co-occurs in four genomes, especially two times in Cma, showing that they should be from the same proto-chromosome, even one being prior to the cucurbit-common whole-genome duplication; this leads to the inference of proto-chromosome 13. Similarly, we inferred proto-chromosome 10.

Section ‘Genomic variation and population structure’:

-Selective sweeps have been investigated using π and Tajima D. How and why the authors selected such metrics among several population genetics methods available to date (Fst, ROD, XP-CLR…among many other approaches) to investigate signatures of domestication and breeding? What are the impact of using π and Tajima D in defining selective sweeps compared to the other approaches?

Response:

We have attempted to use several population genetic methods such as ROD, Tajima D,

XP-CLR to investigate signatures of domestication and improvement. About 74.5% regions overlapped between the results from different methods, using the same cutoff. To improve the accuracy, we first define the regions with the top 10% reduction of nucleotide diversity, and then excluded those without top 50% of XP-CLR scores, as in apple[3] (Response Table 1).

Section 'Candidate genes conferring fruit traits':

-How the candidate genes are selected among the GWAS/QTL intervals and associated improvement/domestication sweeps? Several candidates may probably pass the considered criteria, why and how a single candidate is then presented/selected? How the interval boundaries are defined? How many annotated genes in the intervals? How many gene with selection footprints? How a single gene is finally selected as candidate? Any functional validation (using publicly available resources among the Cucurbitaceae) have been conducted to propose the delivered candidate genes?

Response:

To facilitate the identification of candidate genes responsible for fruit size, we generated RNA-seq data for fruit at three (0, 10 and 20 DAP) developmental stages, for the large (B227) and small (B214) fruited accessions. Differentially expressed genes were then used to identify potential candidate genes for fruit size during domestication and improvement.

In addition, we collected the genes responsible for fruit size in tomato and other cucurbits. By aligning protein sequences of these genes against the genes within sweep and fruit-size related QTL regions, in wax gourd, one orthologous (*Bhi10G0001968*) gene was identified. In addition, a gene with more supporting evidence for a role in fruit enlargement was included in the revised manuscript.

Minor concerns

The authors should make sure that all the data are provided as supplementary datasets;

-OrthoMCl gene families.

-List of orthologous and paralogous genes.

-List of genes with domestication and improvement sweeps.

-List of GWAS and QTL regions and associated annotated genes.

Among many others, so that that "skilled in the art" can reproduce the results and can integrate the delivered data in complementary analyses.

Response:

We have now provided all the data in our revised manuscript.

References

1. Hufford MB, *et al.* Comparative population genomics of maize domestication and improvement. *Nat Genet* **44**, 808-811 (2012).
2. Wu J, *et al.* Diversification and independent domestication of Asian and European pears. *Genome Biol* **19**, 77 (2018).
3. Duan N, *et al.* Genome re-sequencing reveals the history of apple and supports a two-stage model for fruit enlargement. *Nat Commun* **8**, 249 (2017).
4. Xu C, *et al.* A cascade of arabinosyltransferases controls shoot meristem size in tomato. *Nature genetics* **47**, 784 (2015).
5. Li MX, Yeung JM, Cherny SS, Sham PC. Evaluating the effective numbers of independent tests and significant p-value thresholds in commercial genotyping arrays and public imputation reference datasets. *Hum Genet* **131**, 747-756 (2012).
6. Sebastian P, Schaefer H, Telford IR, Renner SS. Cucumber (Cucumis sativus) and melon (C. melo) have numerous wild relatives in Asia and Australia, and the sister species of melon is from Australia. *Proc Natl Acad Sci U S A* **107**, 14269-14273 (2010).
7. de Meeus T, Goudet J. A step-by-step tutorial to use HierFstat to analyse populations hierarchically structured at multiple levels. *Infect Genet Evol* **7**, 731-735 (2007).
8. Tian Gui li ZSp, Song Zi chao, Zhang Song, Cui Jin ying, Miao Han, Gu Xing fang. Genetic Analysis and QTL Mapping of Wax Powder on the Surface of Cucumber Fruit. *Scientia Agricultura Sinica* **48**, 3666 (2015).

Second round of review

Reviewers' comments:

Reviewer #1 (Remarks to the Author):

Relatively limited further experimental data was provided to the current version of the manuscript with respect to the candidate genes classification, prioritization or validation (differential gene expression data between the parental lines on developing fruits was added). This part of the manuscript was adjusted and slightly improved and is now more coherent and focused. While it now better reflecting the message that the combined approaches of genetic mapping and selection signature regions detection can be informative to detect candidate domestication genes, none of the candidates presented pass beyond this level of hypothesis.

Specific comments:

1. Lines 302-309: Bhi10G001538 gene is candidate only based its localization within a domestication sweep region and on differential expression between two large and small-fruited accessions. This, in my view, is still very weak and speculative. In addition, it seems that a fruit size QTL was not mapped in this interval, which further weakens this hypothesis.

Response:

Thanks for your comments. To provide more evidence for the candidate gene *Bhi10G001538*, we analyzed its ortholog (*Csa2G258100*) in cucumber. It was found that *Csa2G258100* was mapped in the QTL interval *fd2.1* for fruit diameter[1]. RNA-seq data of two near isogenic cucumber lines bearing different fruit in length show that this gene is significantly up-regulated in long-fruited line[2] (Response Fig. 1), which is consistent with its role in wax gourd.

In addition, to test the function of SAUR(*Bhi10G001538*) gene in cell expansion in wax gourd, we transiently expressed *35S-MYC-SAUR* and a vector control in cotyledons of wax gourd by agroinfiltration. The expression of MYC-SAUR protein was detected by immunoblotting using anti-MYC antibodies when infiltrated with

OD_{600} at 0.9 (Response Fig. 2a). We further investigated the effects of expression of SAUR protein on the cell size of epidermal pavement cells at 5 days post infiltration. The results revealed that the cell size was significantly larger in cotyledon expressing MYC-SAUR (Response Fig. 2b and 1c). These results provide further evidence that the SAUR gene plays an important role in cell expansion and plant organ size.

For wax gourd, we have not developed a high-efficiency transformation system. Thus, presently, it is not possible to validate the function of this gene using transformation system. In a future project, we plan to validate these genes for wax gourd. In this manuscript, we focus on the novel insights into the ancestral cucurbit karyotype and the genetic basis of wax gourds' diversity through our analyses of these genome data. These new findings have been incorporated into the revised manuscript.

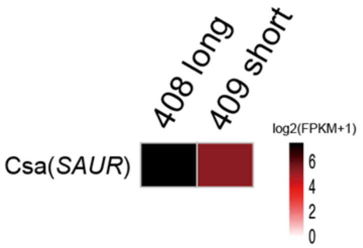

Response Fig. 1 Differential expression of *SAUR* gene in two near isogenic cucumber lines.

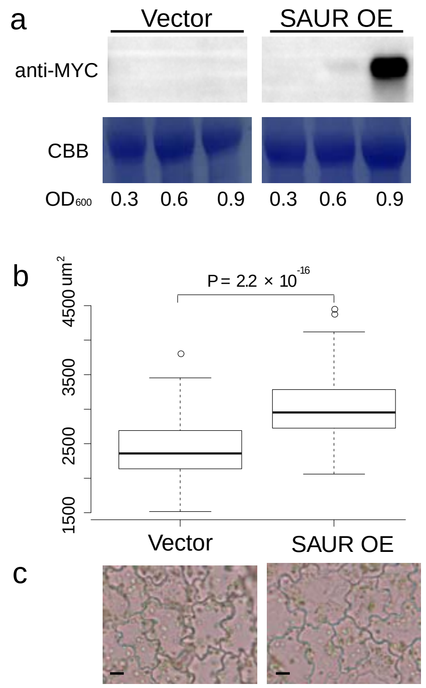

Response Fig. 2 Transient expression of *Bhi10G001538*. **a** Expression profiles of *Bhi10G001538* protein using OD_{600} at 0.3, 0.6 and 0.9, Coomassie brilliant blue (CBB) staining (lower panel) shown as a loading control. **b-c** Boxplots and morphology indicating epidermal pavement cell size of wax gourd cotyledon with *Bhi10G001538* overexpression and empty vector. (OE: overexpression) Bar = 50 µm.

2. Lines 321-366: the gene SlFIN (soloyc11g064850) is presented as a candidate. I Couldn't find it at the provided reference (#27). Also, on line 365 it is stated that it is highly expressed in the fruit (Figure S14). This is not completely correct. The highest expression of this gene according to figure S14 is in the leaf and root.

Response:

We apologize for the error in the reference which, unfortunately, was mis-linked by endnote automatically. This error has been corrected in the revised manuscript. *SlFIN* (*Solyc11*g064850) is expressed in different tissues and at different stages in tomato[3]. We also investigated the *SlFIN* ortholog in other cucurbit species, such as cucumber and melon. In cucumber, the highest expression of *FIN* is in the leaf[4] and for melon it

is in root[5]. But *FIN* is highly expressed in the fruit in all three cucurbit species (Response Fig. 3). *BhiFIN* (*Bhi10G000196*) is significantly down-regulated in the large-fruited accession (Fig. 5c). In addition, *CsaFIN* is down-regulated in the long-fruited accession of two cucumber near isogenic lines with different fruit lengths[2] (Response Fig. 4). To avoid any misunderstanding, we deleted the Supplementary Figure 14. These data also support the sated role of this candidate gene.

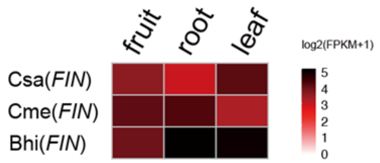

Response Fig. 3. Expression profiles of *FIN* genes in three tissues in three species.

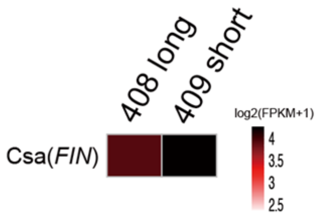

Response Fig. 4. Differential expression of *FIN* gene in two near isogenic cucumber lines.

3. In figure 5c, labels for the developmental stage is required below each column.

Response

Thanks for the suggestion. We added labels for Figure 5c in the revised manuscript.

Reviewer #2 (Remarks to the Author):

The authors have addressed most of my concerns. I still have one major concern left:

In their response, the authors stated that same as in maize and pear studies, genome regions with top 10% nucleotide diversity ratio were selected as selective sweeps in

their original manuscript. I checked the two related papers (Hufford et al. and Wu et al.) and found it is not as described by the authors. The maize one used top 10% XP-CLR score combined with top 50% of reduction in nucleotide diversity and the pear one used top 5% of FST, ROD > 0.5, and bottom 10% of Tajima's D distribution.

In the revised manuscript, the authors use top 10% nucleotide diversity ratio combined with top 50% of XP-CLR score. The authors stated that same criteria was used in the apple study. Again, I checked the apple paper (Duan et al.) and found they used top 10% XP-CLR score combined with top 50% nucleotide diversity ratio, same as the maize one but different from what authors used here for wax gourd. Selective sweeps identified by the authors with the new parameters still occupied a high portion of the genome and could contain quite a lot of false positives. A more stringent criteria is recommended, or use top 10% XP-CLR score combined with top 50% nucleotide diversity ratio, same the one used for maize and apple studies.

Response:

Thanks for the comments. Actually, we identified the sweep regions using both criteria: (1) top 10% nucleotide diversity ratio combined with top 50% XP-CLR score (denoted hereafter as criteria 1) (Response Table1); (2) top 10% XP-CLR score combined with top 50% nucleotide diversity ratio (denoted hereafter as criteria 2) (Response Table2). Comparing the two results we show that they share more than 79.0% (84.3 Mb) domestication and 77.3% (62.1 Mb) improvement regions. All nine mapped QTL intervals for fruit size overlap with the sweeps, of which the major QTL *fw3.1*, on chromosome 3, shows a strong sweep signal using criteria 1, but weak using criteria 2. Therefore, we selected using criteria 1.

Response Table1. Summary of sweep regions in the wax gourd genome with criteria 1.

	Domestication	Improvement

Number of sweep regions	234	168
Max sweep region (Kb)	2,580	2,440
Min sweep region (Kb)	200	200
Average sweep region (Kb)	456	475
Total sweep region length (Mb)	106.7	80.3
Ratio of the assembly (%)	11.60	9.10
Number of genes	3,939	2,251

Response Table2. Summary of sweep regions in the wax gourd genome with criteria 2.

	Domestication	Improvement
Number of sweep regions	227	153
Max sweep region (Kb)	2,360	2,500
Min sweep region (Kb)	200	200
Average sweep region (Kb)	476	490
Total sweep region length (Mb)	108.1	75.1
Ratio of the assembly (%)	11.84	8.23
Number of genes	3,953	2,127

Reviewer #3 (Remarks to the Author):

The article submitted to Nature Communications (manuscript number NCOMMS-19-00378) entitled 'The wax gourd genomes offer insights into the ancestral cucurbit karyotype and the genetic basis of diversity', by Dasen Xie and collaborators delivers the genome sequence of wax gourd and investigates the evolution of the cucurbit genomes, the dynamics of repeated elements, the genomic footprint of domestication and improvement as well as delivers candidate genes driving fruit traits. The article has been revised based on previous concerns and recommendations. Whereas additional analyses performed by the authors respond to previous concerns, please find below additional comments that may improve the current manuscript:

Lines 26-28 page 3: The sentence 'Comparing the genomes of cucumber, melon, watermelon, and bottle gourd revealed that the 12 chromosomes of melon probably

represent the ancestral karyotype of the cucurbit species' could be changed into 'comparing a few available genomes at that time for cucumber, melon, watermelon and bottle gourd previously proposed that the 12 chromosomes of melon may represent the ancestral karyotype of the cucurbit species' (to address that the resolution was not the same in term of comparative genomics in previous analyses).

Response:

Thanks you for your suggestions. We changed the sentence as suggested.

Lines 109-114 page 5: Supplementary Figure 6 proposes a ECH-based re-calibration of the Ks peaks for speciation and duplication. 16.3, 18.1, 26.4 and 36.1 MYA mentioned in the text refer to the uncalibrated or recalibrated values? Why in the Figure 1b the 2 peaks (CCT and ECH) seems both re-calibrated, when, as far as I understand, either it is possible to re-calibrate (the speciation date and associated KS values) with ECH (and then CCT is not calibrated) OR CCT (and then ECH is not calibrated). CCT and ECH cannot be re-calibrated on the same KS plot as they will derive different correction rates?

Response:

Thank you for your comments. Firstly, the time nodes (16.3, 18.1, 26.4 and 36.1 MYA) mentioned in the main text are recalibrated values after the corrections. Secondly, considering that the cucurbits underwent an extra specific polyploidization (CCT) after ECH, we performed a further round correction, at the CCT event, based on the first correction in ECH, and displayed both the ECH and CCT on the same Figure 1b.

Lines 114-119 page 5: The sentence 'In wax gourd, 32 gene families comprising 324 genes exhibited significant expansions ($p<0.01$) relative to their ancestor (Supplementary Data 1). Some of these families were annotated as cytochrome b-c1 complex subunit, zinc finger protein and NBS-LRR resistance genes (Supplementary Table 6, Supplementary Fig. 5). These genes may be associated with the specific

features of wax gourd' seems too vague and would need precision (for example providing the GO for the (10) most expanded families instead of some (arbitrary)).

Response:

We rephrased this sentence to read: "These genes might be a resource for investigating the specific features of wax gourd".

Lines 150-153 page 6: The sentence 'The 12 melon chromosomes were previously considered the most ancestral karyotype in the cucurbits4; however, only five melon chromosomes (chromosomes 2, 8, 9, 10, and 12) were well preserved in the pumpkin genome (Supplementary Fig. 7)' could be changed into: 'The 12 melon chromosomes were previously proposed as the most ancestral karyotype from few cucurbit genomes available at that time; however, only five melon chromosomes (chromosomes 2, 8, 9, 10, and 12) appear to be well preserved in the pumpkin genome (Supplementary Fig. 7)' (to address that the resolution was not the same in term of comparative genomics in previous analyses)

Response:

Thanks you for your suggestions. We changed the sentence as suggested.

Lines 163-186 page 7: This section can be simplified (shorten by half) in three subsections (of the same paragraph):

(1) Entire wax gourd chromosomes preserved in several cucurbit genomes (with melon for Bhi 2, 3, 4, 5, 7, 8, 10 and pumpkin for Bhi2, Bhi3, and Bhi10) defining proto-chromosomes 1-7;

(2) Large patches of wax gourd chromosomes independent in several extant genomes (defining proto-chromosomes 8, 9);

(3) large patches of different wax gourd chromosomes linked in several extant genomes (defining protochromosomes 10 and 13);

Response:

We shortened these sentences as suggested.

Lines 199-202 page 8: the sentence ‘After wax gourd, the melon genome best preserved the ancestral karyotype of cucurbits, with seven melon chromosomes (chromosomes 2, 3, 6, 8, 9, 10, and 12) derived directly from the ancestral ones’ could be changed into ‘After wax gourd, the melon genome best preserved the ancestral karyotype of cucurbits as previously reported4 , with seven melon chromosomes (chromosomes 2, 3, 6, 8, 9, 10, and 12) derived directly from the ancestral ones’ (for consistency with the introduction)

Response:

We changed the sentence as suggested.

Regarding the section devoted to ‘Genomic variation and population structure’, the authors addressed the previous concerns in running and comparing several population genetics methods (with 47.5% of consistency) to investigate signatures of domestication and breeding.

Regarding the section devoted to ‘Candidate genes conferring fruit traits’ the authors conducted RNA-seq experiments (as well as synteny inference with melon genes as suggested by one of the reviewer) to provide ‘functional’ support to the reported candidate genes.

Response:

Thanks you for your comments.

References

1. Pan, Y. et al. QTL mapping of domestication and diversifying selection related traits in round-fruited semi-wild Xishuangbanna cucumber (Cucumis sativus L. var. xishuangbannanesis). *Theoretical and Applied Genetics* **130**, 1531-1548 (2017).
2. Jiang, L. et al. Transcriptomic analysis reveals the roles of microtubule-related genes and transcription factors in fruit length regulation in cucumber (Cucumis sativus L.). *Sci Rep* **5**, 8031 (2015).

3. Shinozaki, Y. et al. High-resolution spatiotemporal transcriptome mapping of tomato fruit development and ripening. *Nat Commun* **9**, 364 (2018).

4. Li, Z. et al. RNA-Seq improves annotation of protein-coding genes in the cucumber genome. *BMC Genomics* **12**, 540 (2011).

5. Yano, R., Nonaka, S. & Ezura, H. Melonet-DB, a Grand RNA-Seq Gene Expression Atlas in Melon (Cucumis melo L.). *Plant Cell Physiol* **59**, e4 (2018).

Third round of review

Reviewers' comments:

Reviewer #1 (Remarks to the Author):

The relevant comments were addressed. no further comments.

Reviewer #2 (Remarks to the Author):

The authors have addressed my concerns.

Reviewer #3 (Remarks to the Author):

The article submitted to Nature Communications (manuscript number NCOMMS-19-00378B) entitled ‘The wax gourd genomes offer insights into the ancestral cucurbit karyotype and the genetic basis of diversity’, by Dasen Xie and collaborators delivers the genome sequence of wax gourd and investigates the evolution of the cucurbit genomes, the dynamics of repeated elements, the genomic footprint of domestication and improvement as well as delivers candidate genes driving fruit traits.

The article has been revised based on previous concerns and recommendations (sun). Whereas additional recommended analyses have not been performed, the text modification have been included in the current version of the manuscript, as detailed below:

Lines 26-28 page 3: Text corrections have been done.

Lines 109-114 page 5: The recommendation have been taken into consideration with the Figure 1b displaying both ECH and CCT recalibration.

Lines 114-119 page 5: No additional analysis have been performed (for example providing the GO for the 10 most expanded families instead of some arbitrary

investigation) as proposed, but text modifications have been included in the revised version of manuscript.

Response:

Thanks for your suggestions. However, only 45 genes of 324 genes in 32 significantly expanded gene families can be assigned GO terms. It makes no sense to conduct additional analysis for the 10 most expanded gene families. So we rephrased this sentence to read: “These genes might be a resource for investigating the specific features of wax gourd”.

Lines 150-153 page 6: Text corrections have been done.

Lines 163-186 page 7: Text corrections have been done.

Lines 199-202 page 8: Text corrections have been done.

I am satisfied by most of the revision performed in the current version of the manuscript.
